# Strategies for understanding the role of cellular heterogeneity in the pathogenesis of lung cancer: a cell model for chronic exposure to cigarette smoke extract

**DOI:** 10.1186/s12890-022-02116-6

**Published:** 2022-09-02

**Authors:** Dong Xia, Jieyi Liu, Juanjuan Yong, Xiang Li, Weidong Ji, Zhiqiang Zhao, Xiaohui Wang, Chen Xiao, Sai Wu, Huaixiang Liu, Heping Zhao, Yun He

**Affiliations:** 1 Department of Toxicology, School of Public Health, Sun Yat-Sen University, Guangzhou, 510080 Guangdong People’s Republic of China; 2 Department of Pathology, Sun Yat-Sen Memorial Hospital, Sun Yat-Sen University, Guangzhou, 510080 Guangdong People’s Republic of China; 3 Zhengzhou Tobacco Research Institute of CNTC, Zhengzhou, 450001 Henan People’s Republic of China; 4 Center for Translational Medicine, The First Affiliated Hospital, Sun Yat-Sen University, Guangzhou, 510080 Guangdong People’s Republic of China

**Keywords:** Cell model, Cell heterogeneity, Malignant transformation, Invasion-phenotype, Chronic exposure

## Abstract

**Background:**

Human tumors are highly heterogeneous at the cellular, molecular, genetic and functional levels. Tumor heterogeneity has tremendous impact on cancer progression and treatment responses. However, the mechanisms for tumor heterogeneity have been poorly understood due to the lack of experimental models.

**Methods:**

This study provides a novel exploration and analysis of the impacts of cellular and molecular heterogeneity of human lung epithelial cells on their malignant transformation following chronic exposure to cigarette smoke extracts.

**Results:**

The ability of cigarette smoke extract (CSE) to cause malignant transformation of the human bronchial epithelial cells (16HBE) is dependent on the sizes of the cells. Epithelial-mesenchymal transition (EMT) plays an important role in this process. Mechanistically, CSE-induced malignant transformation of 16HBE cells was closely linked to the reduced relative telomere length of the larger 16HBE cells, thereby up-regulation of the expression of stemness genes.

**Conclusions:**

These findings provide novel insights for understanding the impact of cellular heterogeneity in lung cancer development. The in vitro transformation model described in this study could be extrapolated to studying the pathogenesis of other malignancies, as well as for mechanistic studies that are not feasible in vivo.

**Supplementary Information:**

The online version contains supplementary material available at 10.1186/s12890-022-02116-6.

## Background

Lung cancer is one of the world’s most common malignancies. Data show that both the prevalence and the mortality of lung cancer have grown in recent years, with high incidence and low survival rates making lung cancer the leading cause of cancer death. Etiological studies have shown that smoking is the leading cause of lung cancer and has become the second most common cause of death worldwide. Nearly 5000 chemicals are found in mainstream tobacco smoke, of which 55 have been evaluated by and identified as “full carcinogenic evidence for experimental animals or humans” by the International Agency for Research on Cancer (IARC). These carcinogens include polycyclic aromatic hydrocarbons, nitrosamines, 1,3-butadiene, ethyl urethane, nickel, chromium, strontium, arsenic, and hydrazine, all of which cause DNA mutation and ultimately lead to lung cancer [1]. Thus, studying the mutagenic effects of tobacco smoke on the human body in order to control and reduce the content of toxic and harmful substances in tobacco and provide accurate data for clinical treatment is an urgent research area. To date, tobacco toxicity evaluation has been conducted using alternatively carcinogenic compound in smoke, such as benzo(a)pyrene [B(a)P] or methylnitrosaminepyridinone (NNK) [2, 3]. However, as tobacco contains multiple carcinogens, this does not fully reflect the interaction of various compounds in tobacco smoke. In addition, in experiments on malignant transformation caused by smoking cigarettes, there have been few results reporting the transformation process, or the molecular biological changes during this process. Therefore, it is necessary to conduct a comprehensive study on the smoke generated by cigarette combustion to explore the mechanism of smoking-induced lung cancer, and to formulate corresponding countermeasures.

Human-derived bronchial epithelial cells are the primary target of smoking-induced lung cancer. In the histopathology of lung cancer, most lung cancers originate from epithelial cells, especially bronchial epithelial cells [4]. In vitro experiments with epithelial cells have the advantages of single processing factors, short cycle, and high sensitivity [5]. 16HBE cells are human bronchial epithelial cells transfected with HPV-16 virus, most of which have diploid karyotypes. They are easy to culture and pass through in vitro, have unlimited growth potential, no spontaneous transformation potential, and do not have malignant transformation characteristics. As such, they are highly suitable for cell transformation experiments [6]. We used 16HBE cells in this study because they originate from the human lung and exhibit similar characteristics and cellular responses to carcinogens as the primary or normal lung cells [7–9]. Moreover, they can be grown continuously in culture, thus allowing the long-term exposure studies that are unfeasible with primary lung cells. These cells are also often used to define conditions under which oncogenes and various agents cause neoplastic transformation [10, 11]. Recently, a research study data show that smoke exposure drives a complex landscape of cellular alterations that may prime the human bronchial epithelium for disease [12]. Another report shows that tobacco smoking increases cell-to-cell heterogeneity, mutational burden and driver mutations. The people who had never smoked had considerably longer telomeres than the people with a history of smoking who had mutational burdens [13]. Quiescent stem cells have been identified through lineage tracing in mouse models and have been shown to occupy a protected niche in submucosal glands and to expand after lung injury [14, 15]. Previous experiments have shown that not all normal human bronchial epithelial cell line 16HBE cell sizes are homogenous. Another study has reported that the proliferation and colony formation ability of epithelial cells with diameter less than 11 μm is significantly higher than those of epithelial cells with diameter 20 μm or more, showing a strong correlation between small sizes of 16HBE cells and high capacity and high amplification colony formation [16]. Moreover, small epithelial cells have similar characteristics to stem cells, proliferate faster than larger epithelial cells, and have higher clone formation rates. Therefore, we sorted cells by size in order to examine 16HBE’s heterogeneity. Because heterogeneity originates in the embryonic stage of lung development, we also needed to use the lung’s key developmental genes during the embryonic stage to study heterogeneity’s role in malignant transformation.

To date, there have little reports on the effects of human lung epithelial cell heterogeneity on the malignant transformation process after chronic CSE exposure, either in vivo or in vitro. Since carcinogenesis is a multi-step process requiring long-term exposure to carcinogens, this information is necessary for evaluating CSE’s epithelial carcinogenic effect.

16HBE, a normal human bronchial epithelial cell line, is a human papilloma virus HPV-16 transfected normal human bronchial epithelial cell line that has become an immortalized bronchial epithelial cell line. Therefore, it has an epithelial phenotype and prolonged passaging without a malignant transformation phenotype [4]. Scholars have studied the relationship between cell sizes and the degree of differentiation, and research has shown that cell size increase leads to an increase in cell differentiation markers [17]. In vitro experimentation provides the best expansion capability and maximum colony formation [18, 19]. We decided to use 16HBE difference in cell sizes to study 16HBE heterogeneity, and to explore whether they have expansion capability and maximum colony formation in vitro, a feature common to all stem cells.

We have developed a chronic exposure cell model in which human lung epithelial 16HBE cells were continually exposed to low doses of CSE in culture over a prolonged time period. During this chronic exposure, we evaluated the cells’ malignant transformation process in vitro. Our results show that chronic exposure to CSE causes malignant transformation in vitro, as well as those larger cells are more likely to transform into cancer cells. The risk factors related to chronic exposure to CSE are a public health concern and should be considered an impetus to implement exposure control strategies. The in vitro transformation model we describe could be used both as a screening assay for the carcinogenic potential of other carcinogens, and for any mechanistic studies which may not be feasible in vivo.

Our results suggest that long-term exposure to CSE leads to malignant transformation of 16HBE cells in vitro. CSE-induced malignant transformation of 16HBE occurs by reducing the relative telomere length of the larger 16HBE cells, thereby causing mutations in cell genes, and up-regulating the expression of related stemness genes. The mass of larger size epithelial cells transforms into cells of stemness ability through EMT which slowly transformed into malignant tumors. These data underscore the dangers of CSE exposure, provide a theoretical basis for evaluating lung cancer hazards and population protection, and identify possible biological targets. The data of malignant transformation process also provide a basis for the early diagnosis of lung cancer.

## Methods

### Preparing the cigarette smoke extract

The cigarette smoke was created with a cigarette smoke machine and then filtered using a Whatman Cambridge filter. The adsorbate was dissolved in DMSO via manual agitation, and the sterile cigarette smoke extract solution was obtained via centrifugation and filtration to produce final concentrations for extracting 10 mg/mL and 40 mg/mL.

### Cell culture

The human bronchial epithelial cell line 16HBE and 293 T cells, obtained from Professor Ji Weidong (Center for Translational Medicine, First Affiliated Hospital of Sun Yat-sen University), cultured in DMEM (GIBCO, USA) containing 10% heat-inactivated fetal bovine serum (FBS; GIBCO), 10,000 U/mL penicillin, 10 mg/mL streptomycin (GIBCO), and 4 mM l-glutamine. The cells were grown in a humidified atmosphere composed of 95% air and 5% CO2 at 37 °C. We used cell STR and mycoplasma contamination tests to authenticate the 16HBE cell line was not a contaminating cell line in September 2017. The cell line DNA typing found a completely matched 16HBE cell line in the ATCC database (cell number is CRL-2741) (Additional files 1, 2). No multiple alleles were found in this cell line.

### Sorting 16HBE cells by size

**Fig. 1:**
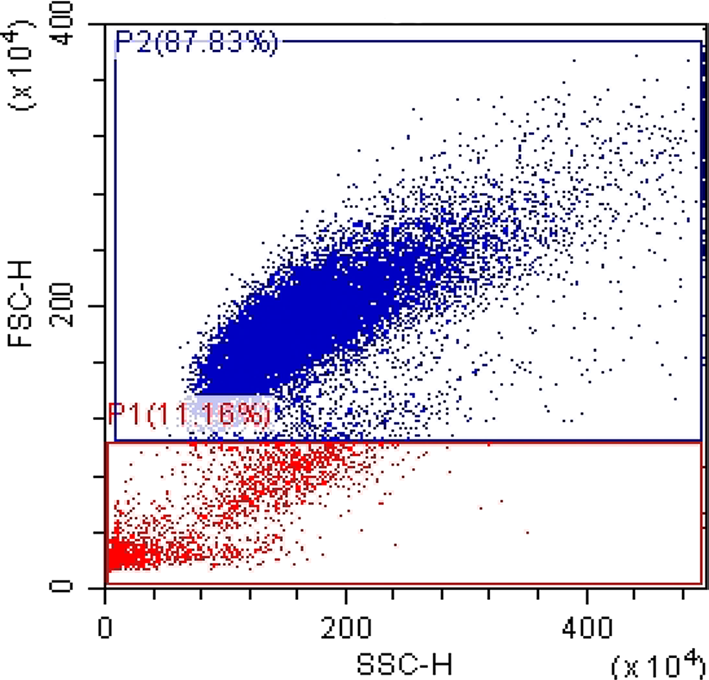
The flow cytometer’s sorting range

16HBE cells were sorted by flow cytometer according to the sample’s sizes at a ratio of approximately 9:1 (as shown in Fig. 1). By setting and adjusting the FSC threshold, most of the interference of cell fragments, bubbles and laser noise (all belonging to the area of FSC low) are excluded from the analysis area. In addition, we further removed cell debris and dead cells by PBS washes and centrifugation. The large cell area was designated as P2, while the small cell area was designated as P1. The two cell sizes were cultured separately. We labeled the bulky cells 16HBE-B, and the small cells 16HBE-S.

### Chronic CSE exposure alters cell morphology and growth patterns

Exposure groups: the human lung epithelial cells 16HBE-B and 16HBE-S in exposure group were exposed to 40% IC50 concentration of CSE in culture and passaged weekly. The CSE-treated cells’ morphological changes after 20 weeks of exposure was observed. The first passage cells were identified as CSE 1 (C1) generation.

Negative controls groups: the DMSO was added in 16HBE-B and 16HBE-S negative group as same volume as exposure group, and the operation is the same as the exposure group. The first passage cells were identified as DMSO 1 (D1) generation.

### Cell proliferation and half maximal inhibitory concentration assays

To determine whether chronic CSE exposure affected cell growth characteristics, we compared the 16HBE-B and 16HBE-S cells’ proliferative rates with a CCK-8 cell proliferation assay (DOJINDO, Tokyo, Japan). The 16HBE cells in a 96-well plate at a concentration of 5 × 10^3^ per well in DMEM supplemented with 10% FBS were seeded. The cells were incubated for 24 h, followed by incubation for 0, 12, 24, 48, and 72 h. At each point, the cells were incubated with CCK-8. Four hours later, absorbance at 450 nm was recorded using an automatic microplate spectrophotometer (Biotek-ELX800, Vermont, USA), and a cell growth curve was drawn according to the data.

We administered CSE to the 16HBE cells and evaluated the IC50 in 16HBE cell lines with a CCK-8 assay. 16HBE cells were seeded at 15 × 10^3^ cells/well in a 96-well plate and incubated for the indicated times (24, 48 and 72 h) at 37 °C. At the end of the selected experimental time, CCK-8 solution was added to all wells of assay and measured the optical density (OD) of each well with an automatic microplate spectrophotometer equipped with a 450 nm filter. The cells’ viability in response to treatment with tested compounds was calculated as: % viable cells = [OD (450–650 nm) experimental group/OD (450–650 nm) negative control] × 100. IC50 was determined using Prism 5.0 (GraphPad Software Inc.). The experiment was repeated three times independently. The IC50 values are presented as means ± SEM of at least three independent experiments conducted in triplicate.

### Fluorescent lentivirus packaging and infection

293 T cells were subcultured to about 70% confluence for transfection. Then, we mixed the lentivirus fluorescent plasmid pMD2.G, psPAX2, CaCl2 transfection reagent with pLVX-IRES-ZsGreen1 or pLVX-IRES-tdTomato respectively. This mixture was then added into the culture dish. For each 6 cm dish, the following transfection mixture was prepared: 2nd mix 1 ug/μL (composition: pspax2, 0.75 ug/μL; pMD2G, 0.25 ug/μL). The composition of packing mix was as follows: lenti DNA: 2nd mix = 1:1. When transfecting, lenti DNA (8 ug) and 2nd mix (8 mL) were used in 10 cm dishes. The preparation of transfection system generally does not exceed 10% of the culture medium system. The lentivirus 800 μL transfection system steps were as follows: 447.4 μL double distilled water and 3 μL mix of the packing mix plasmid were combined. 49.6 μLCacl2 (2 m) was then injected into the bottom of the tube, and finally 2 × HBS 400 μL was added along the pipe wall. It was immediately mixed with a vortex oscillator for 30 s, then left to stand at room temperature for 20 min. After a small part of cell culture medium was absorbed and gently mixed with the transfection system, it was added to 293 T cells dropwise and gently mixed.

After transfection for 12 h, the infected culture medium of 293 T cells was sucked and 8 mL fresh complete culture medium was added, and the culture dish was incubated at 5% CO2 and 37 °C. We collected and mixed the supernatant after 24 h and 36 h as virus solution, respectively. The culture dish with 70% fusion 16HBE-B and 16HBE-S cells was added 5 mL virus solution and 5 mL fresh complete culture medium and cultured for 6–8 h. Then repeatedly infected with a new virus solution and culture medium. After 12 h of infection, the culture medium containing lentivirus was replaced with normal culture medium. The fluorescence was observed under an inverted fluorescence microscope to detect the efficiency of lentivirus infection of target cells. The cells were detected expression rates of ZsGreen and tdTomato with flow cytometry.

### Cell cycle detection

After CSE administration, the cells were digested with 0.25% trypsin–EDTA digestive solution and transferred to a centrifuge tube. Then, cells were centrifuged at 1000 r min^−1^ for five minutes. Next, cells were washed twice with 3 mL of PBS, centrifuged at 1000 r min^−1^ for five minutes, and then resuspended by new PBS solution. The above PBS cell suspension was added to 5 mL of pre-cooled 70% ethanol at 4 °C and was sealed at 4 °C overnight. Then, the cells were collected via centrifugation at 1200 r min^−1^ for 5 min and washed twice with PBS. We added 0.5 mL of DAPI to each tube, dyed them for 15 min, resuspended by new PBS solution, and passed them through a 300 μm nylon mesh. After the test tube had been shaken slightly, we detected the cells’ fluorescent density with a flow cytometer (Beckman CytoFLEX S). We used Modfit LT (Verity Software House) as our analysis software. The experiment was repeated three times independently, and the results were taken as the average. We conducted one-way analysis of variance (one-way ANOVA) for each data group, and the differences between different generations cell cycle were statistically significant when *P* ≤ 0.05.

### Determining relative telomere length and mtDNA copy number

Blood genomic DNA was extracted according to the instruction of Guangzhou Magen Biotechnology of HiPure Tissue & Blood DNA Kit. Design telomeres (Tel), mitochondrial single copy gene ND-1 and internal reference gene 36B4 primers, primer sequences and final concentrations are shown in Table 1. The sequence of real-time qPCR primers was searched in the BLAST database of NCBI, and designed with primer5. We used a 25 μL PCR reaction and included 12.5 μL of 2 × UltraSYBR mixture, 25 ng of genomic DNA, and corresponding sizes of primers and sterile double-distilled water. Each sample was set with three replicate wells. The detective machine is Thermo fisher QuantStudio 7 Flex Real-Time PCR Systems. The target gene was identical to the reference gene’s PCR reaction conditions. The amplification’s thermal profile included the following: Begin the Taq enzyme activation step at 95 °C for 10 min, followed by a PCR protocol for 40 cycles consisting of 95 °C for 15 s, annealing at 60 °C for 1 min, 95 °C for 15 s, 60 °C for 1 min, 95 °C for 15 s, and 60 °C for 15 s. To calculate the relative telomere length and the relative mitochondria copy number, the relative quantification method was used. After obtaining CT values for different genes in each sample, we calculated the ratio of the target gene to the CT value of the single copy gene (T/S): [2 Ct (Tel) /2 Ct (36B4)]-1 = 2^−ΔCt^. Then we divided each sample’s T/S value by the average of the T/S of the control group to obtain the relative T/S value: 2^−ΔCt /2−ΔCt(control)^ = 2^−ΔΔCt^. When the relative T/S = 1, this indicated that the target gene’s expression in the experimental group was identical to that of the control group. When the relative T/S > 1, this indicated that the target gene’s expression was higher in the experimental group than in the control group. The experiment was repeated three times independently, and the results were taken as means ± SD.

**Table 1:** Sequence of real-time qPCR primers of telomere length and mtDNA copy number

Gene name	Primer	Oligo primer sequence
ND-1	F	5′- TCTCACCATCGCTCTTCTACT -3′
	R	5′- AGGCTAGAGGTGGCTAGAATAA -3′
*TEL*	F	5′-CGGTTTGTTTGGGTTTGGGTTTGGGTTTGGGTTTGG GTT V-3′
	R	5′-GGCTTGCCTTACCCTTACCCTTACCCTTACCCTTACCT -3′
*36B4*	F	5′- CAGCAAGTGGGAAGGTGTAATCC -3′
	R	5′- CCCATTCTATCATCAACGGGTACAA -3′

### Invasion assays

We put 100 μL of the diluted Matrigel into the upper chamber of a 24-well transwell, incubating the transwell at 37 °C for at least 4 h for gelling. Then, we resuspended the cells in media containing 1% FBS at a density of 10^6^ cells/mL, and then gently washed gelled Matrigel with warmed serum-free culture media. We added 100 μL of the cell suspension into the Matrigel and filled the lower chamber of the transwell with 600 μL of culture media containing 5 μg/mL fibronectin, which served as an adhesive substrate. We incubated the plate with transwell at 37 °C for 20 to 24 h. Then, transwell were removed from the 24-well plates and stained with Diff-Quick solution. Non-invaded cells were removed from the top of the transwell with a cotton swab, and the invaded cells were counted under a light microscope. The experiment was repeated three times independently, and the results were taken as means ± SD.

### Detecting mRNA expression levels by real-time qPCR

We selected lung development and lung squamous cell carcinoma-related stem genes and related molecules of the epithelial-mesenchymal transition pathway for detection according to previous studies on the malignant transformation of CSE cells in phenotype change and differentiation [20–25]. Therefore, we selected the SOX2, SOX4, SOX9, KRT5, KRT14, NANOG and PD-L1 genes as our research objects.

The total RNA was extracted via the Trizol method, RNA concentration was measured, and cDNA was synthesized through reverse transcription (Table 2). The sequences of real-time qPCR primers were searched in the BLAST database of NCBI and designed with primer5.Reaction system configuration was guided by the instructions from the real-time qPCR kit used. Real-time qPCR reaction conditions were outlined as follows: pre-denaturation at 95 °C for 30 s; 1 cycle of 95 °C for 10 s, 60 °C for 30 s; 40 cycles of 95 °C for 15 s, 60 °C for 60 s, 95 °C for 15 s. See Table 2 for the primer sequence. Relative expression of the target gene was calculated using ${2}^{{ - \Delta {\text{CT}}}}$. ΔCT value = (treatment group target gene CT value—treatment group GAPDH CT value)—(control group target gene CT value—control group GAPDH CT value). The experiment was conducted in triplicate. See Table 2 for the real-time qPCR primer sequence.

**Table 2:** Sequence of real-time qPCR primers of stemness gene

Gene name	Primer	Oligo primer sequence
β-actin	F	5′- CATGTACGTTGCTATCCAGGC -3′
	R	5′- CTCCTTAATGTCACGCACGAT -3′
*SOX9*	F	5′- AGCGAACGCACATCAAGAC -3′
	R	5′- CTGTAGGCGATCTGTTGGGG -3′
*SOX2*	F	5′- GTTCTAGTGGTACGGTAGGAGCTTTG -3′
	R	5′- TTTGATTGCCATGTTTATCTCGAT -3′
*Nanog*	F	5′- CCAGCTGTGTGTACTCAATGATAGATTT -3′
	R	5′- TTCTGCCACCTCTTAGATTTCATTC -3′
*SOX4*	F	5′- AGCGACAAGATCCCTTTCATTC -3′
	R	5′- CGTTGCCGGACTTCACCTT -3′
*PD-L1*	F	5′- GCTGCACTAATTGTCTATTGGGA -3′
	R	5′- AATTCGCTTGTAGTCGGCACC -3′
*Krt5*	F	5′-AGGAGTTGGACCAGTCAACAT -3′
	R	5′- TGGAGTAGTAGCTTCCACTGC-3′
*Krt14*	F	5′-TGAGCCGCATTCTGAACGAG-3′
	R	5′-GATGACTGCGATCCAGAGGA-3′

### Soft agar colony formation experiments

0.5% agar was prepared for the bottom layer, and 0.35% agar was prepared for the top layer. 0.5% soft agar was poured into a 6-well cell culture plate at 4 mL of solution per well. After solidification at room temperature, it was set it at 2 ~ 8 °C for storage, then balanced it in a 37 °C incubator for > 30 min before use. Then, we added 5000 cells and 4 mL 0.35% soft agar to a sterile tube, mixed it and spread it on the bottom layer of agar at 2 mL per well, for duplicate wells. After solidification at room temperature, it was incubated at 37 °C for 14 to 21 days. After continuous culture for about 16 days, we observed it under a microscope, counted the colonies larger than 10 cells, and calculated the cell clone formation rate (cell clone formation rate = average number of cell clones per well/Number of cells added × 100%). This experiment was repeated three times independently.

### Western blot analysis

We examined protein expression with SDS-PAGE and extracted total intracellular proteins from the cells by freezing and thawing in lysis buffer containing protease inhibitors. Protein content was estimated according to the Biorad protein assay (BIO­RAD). We loaded the samples (20 μg proteins) onto denaturing polyacrylamide gel and separated them with SDS-PAGE. The separated proteins were then transferred electrophoretically to nitrocellulose membranes (Immobilon-NC, Millipore). Membranes were blocked with 5% nonfat stemness milk in TBS-Tween 20 (0.1% v/v) and then incubated overnight at 4 °C with the primary antibodies. Proteins were visualized with an enhanced chemiluminescence detection system (Amersham Pharmacia Biotech) after incubation overnight at 4 °C with the primary antibodies E-cadherin (mouse monoclonal; 1:1000, Proteintech) and GAPDH (Mouse polyclonal 1:20,000; Proteintech). Then, the membranes were incubated at room temperature with an appropriate secondary goat anti-mouse antibody (1:5000; Sigma-Aldrich). We detected immunoreactive protein bands through their chemiluminescence using enhanced chemiluminescence reagents (ECL; Amersham). The blots were exposed and analyzed to Las4000 (GE Healthcare Life Sciences). The experiment was repeated three times independently, and the results were taken as means ± SD. Because other proteins were also detected in this experiment, the whole membrane was cut and incubated with different primary antibodies. Additional file 8 and Additional file 9 show the original images of all blots with membrane edges visible.

### Patients and tissue specimens

In this study, we collected 30 clinical and pathological specimens of lung squamous cell carcinoma admitted to Sun Yat-sen Memorial Hospital from June 2018 to January 2019. The specimens had been diagnosed as lung squamous cell carcinoma by pathologists. All 30 patients had at least a 20-year history of smoking and were aged between 52 and 76 years, with an average age of 65 ± 5 years. All 30 patients with lung cancer were newly diagnosed and had not received either radiotherapy or chemotherapy before the specimens were collected. We also collected normal tissue adjacent to the tumors. Other exclusion criteria included severe medical complications, advanced age, refusal of surgery, and tumors in other parts of the body (Additional file 10, Additional file 11).

### Immunohistochemistry

We prepared the paraffin-embedded sections with 4 μm for IHC staining to be performed with an SP immunohistochemistry kit (Nanjing Jiancheng Bioengineering Institute). We detected specific target proteins with a 3.3′-diaminobenzidine peroxidase substrate kit (Nanjing Jiancheng Bioengineering Institute). Table 3 shows the primary antibodies used in this study, including their respective dilutions, immunostaining methods, and sources. We incubated the slides with primary antibodies overnight at 4 °C, and then washed the samples with phosphate-buffered saline (PBS). The experiment was repeated three times independently, and the results were taken as means ± SD. We used PBS in place of the primary antibody as a negative control.

**Table 3:** The primary antibodies used in this study

Primary antibody	Secondary antibody
Rabbit Anti-Cytokeratin 5 antibody [EP1601Y]—Cytoskeleton Marker (ab52635)	Goat Anti-Rabbit IgG H&L (HRP) (ab205718)
Mouse Anti-Cytokeratin 14 antibody [LL002] (ab7800)	Goat Anti-Mouse IgG H&L (HRP) (ab205719)
Rabbit Anti-Vimentin antibody [EPR3776]—Cytoskeleton Marker (ab92547)	Goat Anti-Rabbit IgG H&L (HRP) (ab205718)
Rabbit Anti-EpCAM antibody [EPR20532-225] (ab223582)	Goat Anti-Rabbit IgG H&L (Alexa Fluor® 488) (ab150077)
Rabbit Anti-PD-L1 antibody [73–10] (ab228415)	Goat Anti-Rabbit IgG H&L (Alexa Fluor® 488) (ab150077)
Rabbit Anti-SOX2 antibody (ab97959)	Goat Anti-Rabbit IgG H&L (HRP) (ab205718)

### Statistical analysis

Representative results from Western blots from a single experiment are presented, as additional experiments yielded similar results. We normalized the optical density of the protein bands detected by Western blotting against tubulin levels. Statistical comparisons between groups were conducted using one-way ANOVA or an unpaired, two-tailed t-test comparing two variables. Differences were considered significant if *P* < 0.05. The results of the invasion assays are expressed as the means for at least five independent experiments (± S.E.M.). The RT­PCR data for mRNA expression are representative of at least three independent experiments and include the means ± S.E.M. of technical triplicates. Statistical significance was confirmed by two-sided Student’s t-tests (according to a normal distribution), and all statistically significant *P* ≤ 0.05 are reported either in the manuscript or in the figure legends.

## Results

### Sorting different sizes of 16HBE cells by FACS (fluorescence activated cell sorting)

We used the sorting flow cytometer to separate the 16HBE cells according to sizes at a proportion of approximately 9:1 (as shown in Fig. 1). The forward angle scattering light (FSC) represents the cells’ relative size and surface area, and the side angle scattering light (SSC) represents the cells’ granularity and the relative complexity of the intracellular organelles. The large cell area was designated as P2. The small cell area was designated as P1. According to the FSC-H standards, we collected 11.2% of the cells from area P1 and 87.8% from area P2. We cultured the two cell groups separately, labeling the large cells 16HBE-B and the small cells 16HBE-S.

### Chronic CSE exposure inhibits cell growth in different sizes 16HBE cells

**Fig. 2:**
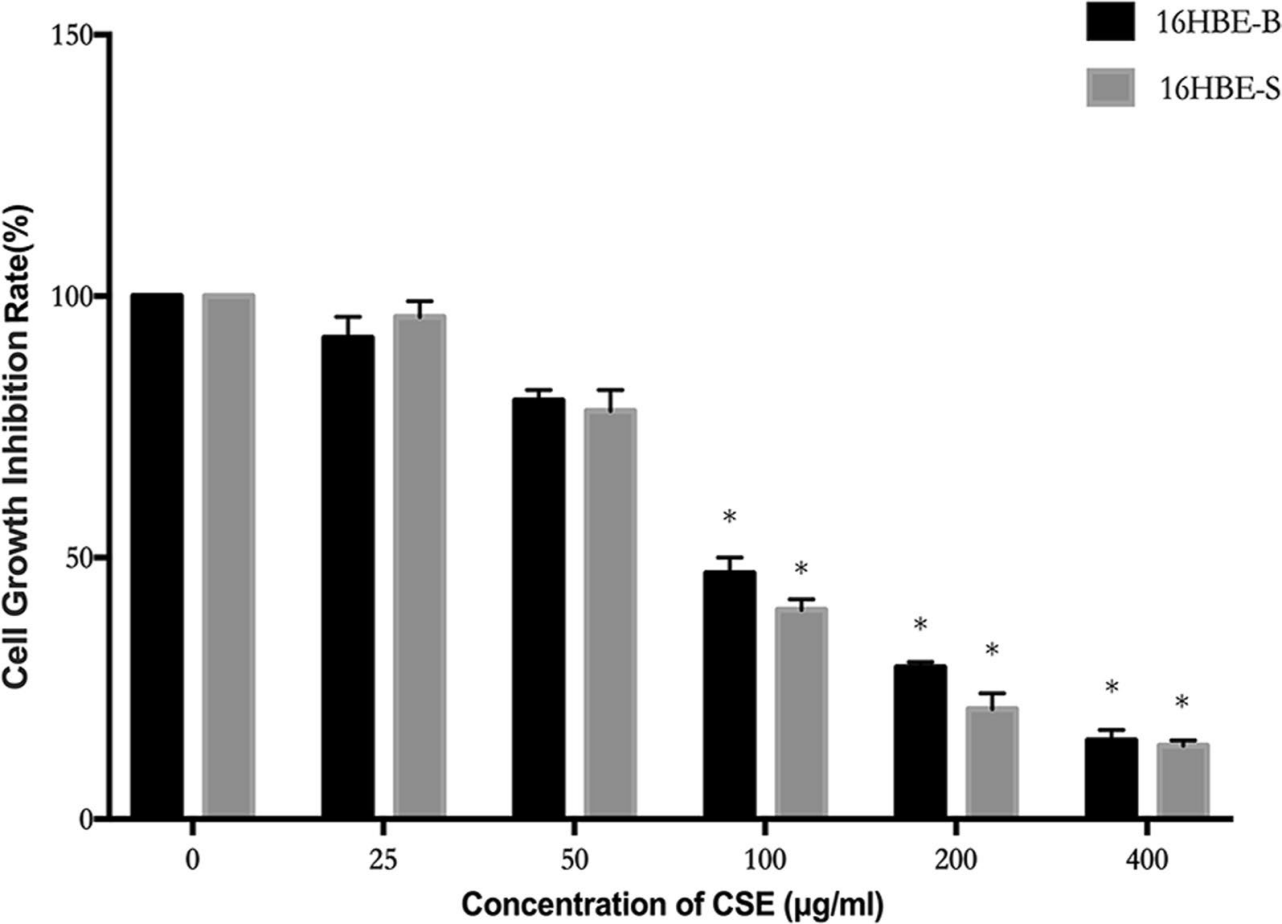
CSE growth inhibition rate on 16HBE-B and 16HBE-S. *P ≤ 0.05; versus 0 μg/mL; the cell inhibition rate is statistically different from 0 μg/mL

As shown in Fig. 2, we combined 0, 25, 50, 100, 200, and 400 μg/mL cigarette smoke extract with 16HBE-B cells and 16HBE-S cells for 48 h. The cells showed different degrees of inhibition. According to the data analysis, the IC50 value of the CSE used was 120 μg/mL, and we used 40% IC50 concentration as the toxic agent for malignant transformation of our cells, i.e., 50 μg/mL (Additional file 3).

### Chronic CSE exposure increases 16HBE-B cell proportion but decreases 16HBE-S cell proportion across generations

**Fig. 3:**
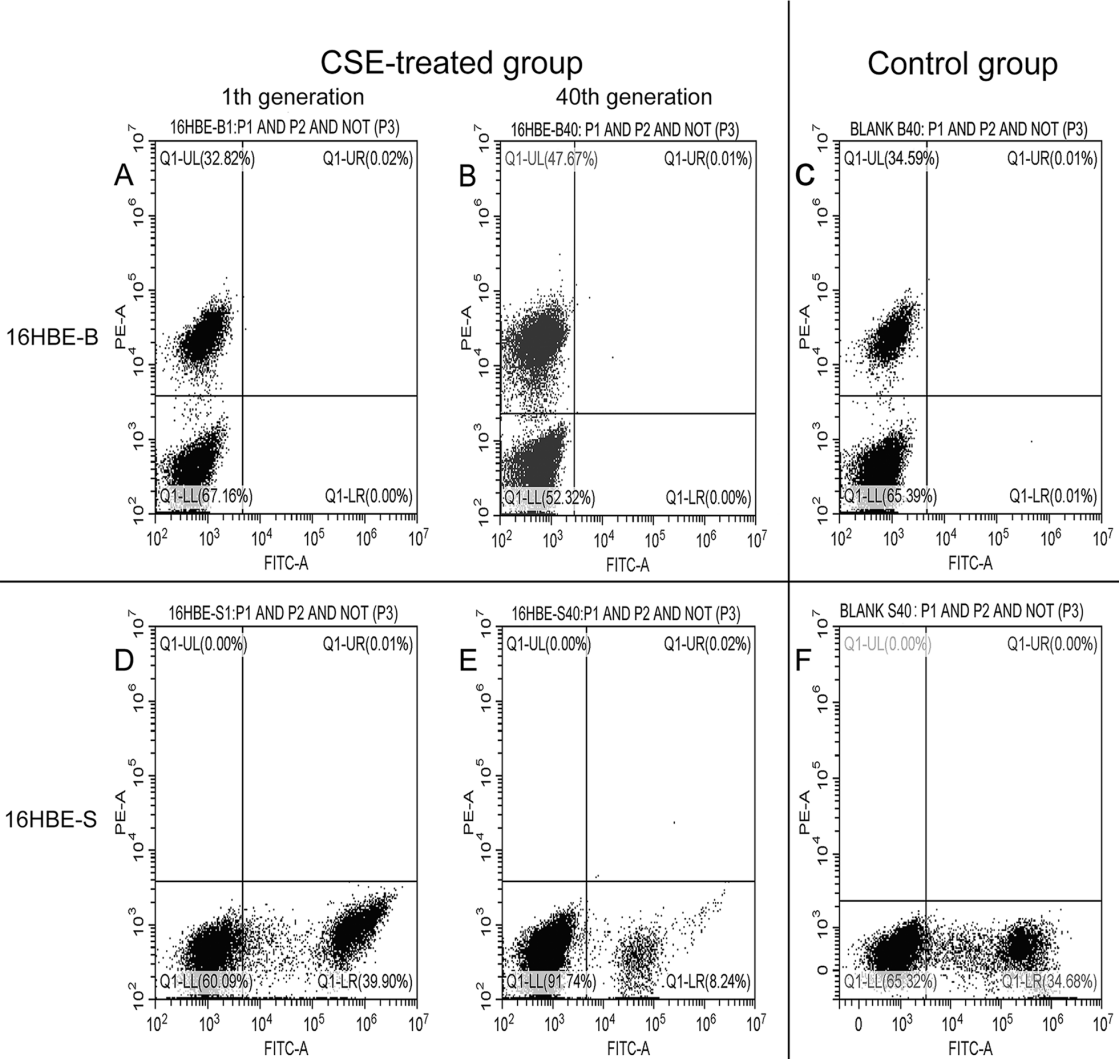
Flow cytometry figure of fluorescence percentage in 16HBE-B and 16HBE-S cells. **A** The proportion of fluorescence positive cells of 16HBE-B first generation. **B** The proportion of fluorescence positive cells of 40th passage 16HBE-B cells with CSE exposure. **C** The proportion of fluorescence positive cells of 40th passage 16HBE-B cells’ normal control group. **D** The proportion of fluorescence positive cells of 16HBE-S first generation. **E** The proportion of fluorescence positive cells of 40th passage 16HBE-S cells with CSE exposure. **F** The proportion of fluorescence positive cells of 40th passage 16HBE-S cells’ normal control group

We used flow cytometry to detect the tdTomato red fluorescence (16HBE-B cells) and ZsGreen green fluorescence (16HBE-S cells) expression levels (as shown in Fig. 3). The results showed that when compared with the control group, the proportion of 16HBE-B red fluorescence cells in the CSE-treated group increased from 32.82% in the C1 generation to 52.17% in the C40 generation. Moreover, the proportion of 16HBE-S green fluorescence cells in the CSE-treated group decreased from 39.90% in the C1 generation to 8.24% in the C40 generation.

### Chronic CSE exposure alters morphology of 16HBE-B and 16HBE-S

**Fig. 4:**
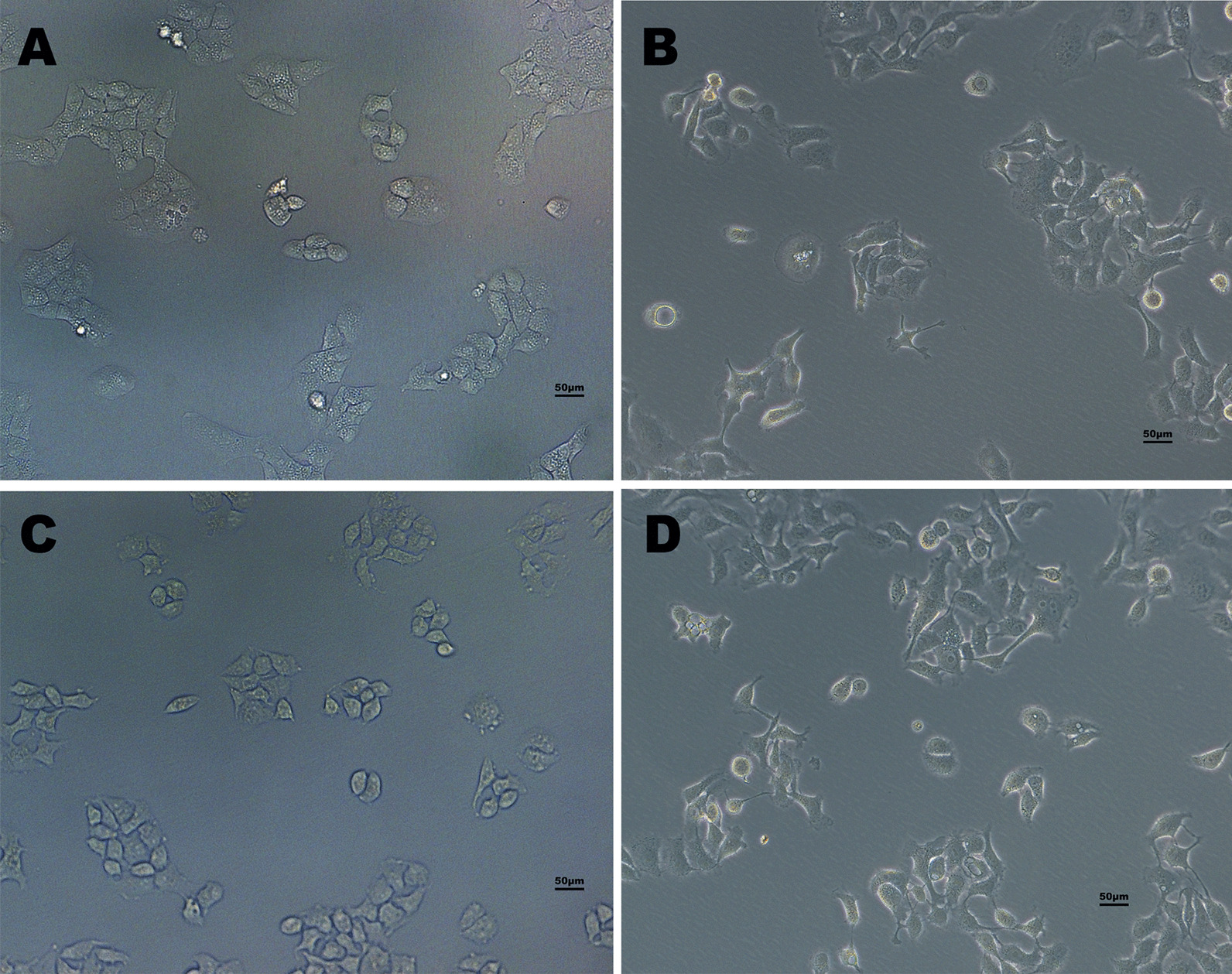
Morphological changes in 16HBE-B and 16HBE-S at passage 40 after CSE treatment. **A** 16HBE-B control group. **B** 16HBE-B exposure group. **C** 16HBE-S control group. **D** 16HBE-S exposure group

As shown in Fig. 4, when 16HBE-B cells and 16HBE-S cells were exposed to CSE for 40 generations, we observed changes in morphology and growth characteristics among both the cells exposed to CSE and the control group. Compared with the control group, the cells in the 40-generation group showed heteromorphism, and the appearance of oval cobble epithelial cells gradually became fusiform. The cell gap also increased. Many cells showed spindle-shaped fibroblast-like morphological changes, and gradually lost the original paving-stone cell growth morphology. The morphological changes in the 16HBE-B cells were more obvious. Slightly less morphological change occurred in the 16HBE-S cells than in the 16HBE-B cells, and there was no obvious visual difference.

### Chronic CSE exposure induces cell growth rate and doubling time changes in variegated 16HBE cells

**Fig. 5:**
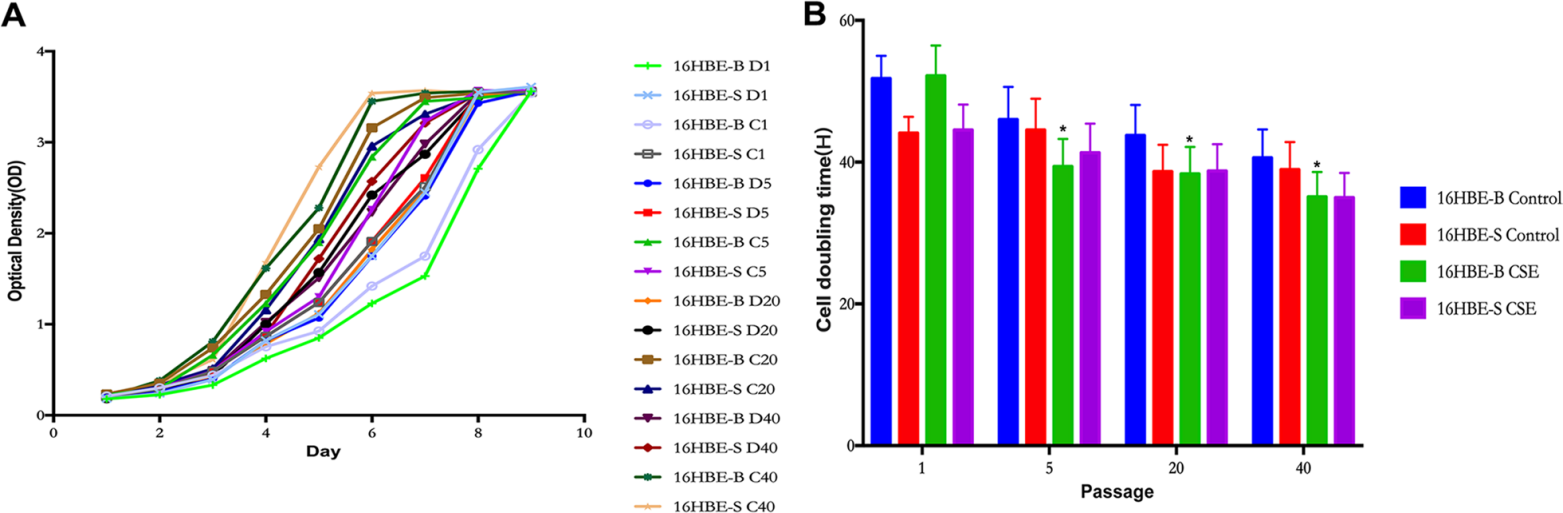
Growth curve and doubling time of 16HBE-B cells and 16HBE-S cells of different generations. **A** 16HBE-B/16HBE-S control group and exposed group cell growth curve of different generations. **B** 16HBE-B/16HBE-S control group and exposed group doubling time of different generations. *: *P* ≤ *0.05*, n = 3, versus 16HBE-S CSE Passage 1

As shown in Fig. 5, we measured the growth curves and calculated the doubling times of the cells in the 16HBE-B cells, the D1, D5, D10, D20, D30, D40 and the C1, C5, C10, C20, C30, C40 generations of the control group and the CSE-treated group. We first compared the cell growth curve and doubling time of the same cell type in different control groups and in the exposed group. The results showed that the D1 generation’s proliferation capacity was the lowest, while that of the C40 generation was the highest. Meanwhile, there was a change in the doubling time, but it was not statistically significant. The proliferation ability of the 16HBE-B and 16HBE-S cells was higher than that of the control cells (Additional file 4).

As shown in Fig. 5A, the groups with the strongest proliferation ability were 16HBE-B in the C40 generation, and the groups with the weakest proliferation ability were 16HBE-B in the D1 generation. The three groups of strongest proliferative ability in 16HBE-B cells were C40, C30 and C20. The three minimum proliferative groups were 16HBE-B D1, C1 and D5. 16HBE-S cells had the strongest proliferative ability in the group of 16HBE-S C40, C30 and C10. 16HBE-S D1, D5 and D10 were the least proliferative groups. Then we compared the growth curves of different groups of cells in each generation. In each generation, the proliferation ability of infected cells was higher than that of control cells. The proliferation ability of 16HBE-S cells in the control group was higher than that of 16HBE-B cells in each generation, but there was no significant difference between 16HBE-S cells and 16HBE-B cells in the exposed group.

As shown in Fig. 5B, in the control group compared with the first-generation cell doubling time, the other generations’ cell doubling time reduction difference was not statistically significant. In the 16HBE-B CSE group, compared with the doubling time of the first-generation cells, the difference of doubling time from the 5th generation cells was statistically significant. In the 16HBE-S CSE group compared with the first-generation cell doubling time, the other generations’ cell doubling time reduction difference was not statistically significant. Compared with the control group, the doubling time of the CSE group was shorter than that of the control group, but the difference was not statistically significant.

### Chronic CSE exposure alters cell cycle in variegated sizes of 16HBE cells

As shown in Table 4, cells in the 16HBE-B exposed group decreased more in the G1 phase and increased in the S phase and G2 phase with an algebraic increase. The results of the chi-square test showed that there was no significant difference in cyclical change between the first generation and the different algebraic cells in the 16HBE-B control group. Compared with the first generation, the change in the cell cycle was statistically significant. Compared with the control group, the cell cycle change in the same generation was statistically significant in the 40th generation.

**Table 4:** The cell cycle of different generations in 16HBE-B control group and CSE-exposed group

16HBE-B passage	Control group			CSE group			Chi-square	*P*
	G1 (%)	S (%)	G2 (%)	G1 (%)	S (%)	G2 (%)		
1	74.99	17.7	7.31	75.59	18.13	6.28	0.058	0.809
5	78.94	16.42	4.64	80.87	12.32	6.80	0	0.999
10	71.07	21.79	7.14	75.45	19.14	5.41	0.657	0.417
20	70.22	24.68	5.10	63.80	28.20	8.00	1.098	0.293
30	64.16	24.69	11.15	56.67^**^	32.33^**^	11.00^**^	0.521	0.470
40	61.87	28.46	9.67	43.28^**ΔΔ^	41.61^**ΔΔ^	15.11^**ΔΔ^	5.878	0.015

*P ≤ *0.05, n* = *3;* **P ≤ *0.01, n* = *3 *versus* P1* CES Group; P: CSE Group versus Control Group

^Δ^*P* ≤  *0.05, n* = *3,* ^ΔΔ^*P* ≤ *0.01, n* = *3 *versus CSE Group versus Control Group

As shown in Table 5, cell counts in the 16HBE-S group increased, cell counts in the G1 phase decreased, and the cell counts in the S phase and G2 phase increased. A chi-square test showed no significant difference in cyclical change between the first generation and the different algebraic cells in the 16HBE-S control group. Compared with the first generation, the cell cycle change was statistically significant. Compared with the control group, the cell cycle change in the same generation was statistically significant in the 30th generation.

**Table 5:** The cell cycle of different generations in 16HBE-S control group and CSE-exposed group

16HBE-S passage	Control group			CES group			Chi-square	*P*
	G1 (%)	S (%)	G2 (%)	G1 (%)	S (%)	G2 (%)		
1	77.34	17.66	5.00	77.85	14.15	8.00	0.0595	0.807
5	81.37	15.92	2.70	84.21	11.36	4.42	0.167	0.682
10	80.64	13.98	5.37	82.18	14.93	2.89	0.177	0.674
20	78.81	15.26	5.94	64.92	27.08	8.00	3.483	0.062
30	69.33	22.67	8.00	56.39^**Δ^	29.07^**Δ^	14.54^**Δ^	4.168	0.041
40	66.32	22.68	11.00	43.17^**Δ^	35.38^**Δ^	21.45^**Δ^	9.565	0.002

*P ≤ *0.05, n* = *3;* **P ≤ *0.01, n* = *3 *versus* P1* CES Group; *P*: CSE Group versus Control Group

^Δ^*P* ≤ *0.05, n* = *3;* ^ΔΔ^*P* ≤ *0.01, n* = *3 *versus CSE Group versus Control Group

### Chronic CSE exposure induced relative telomere length, and the mitochondrial DNA copy number changes in different sizes of 16HBE cells

**Fig. 6.**
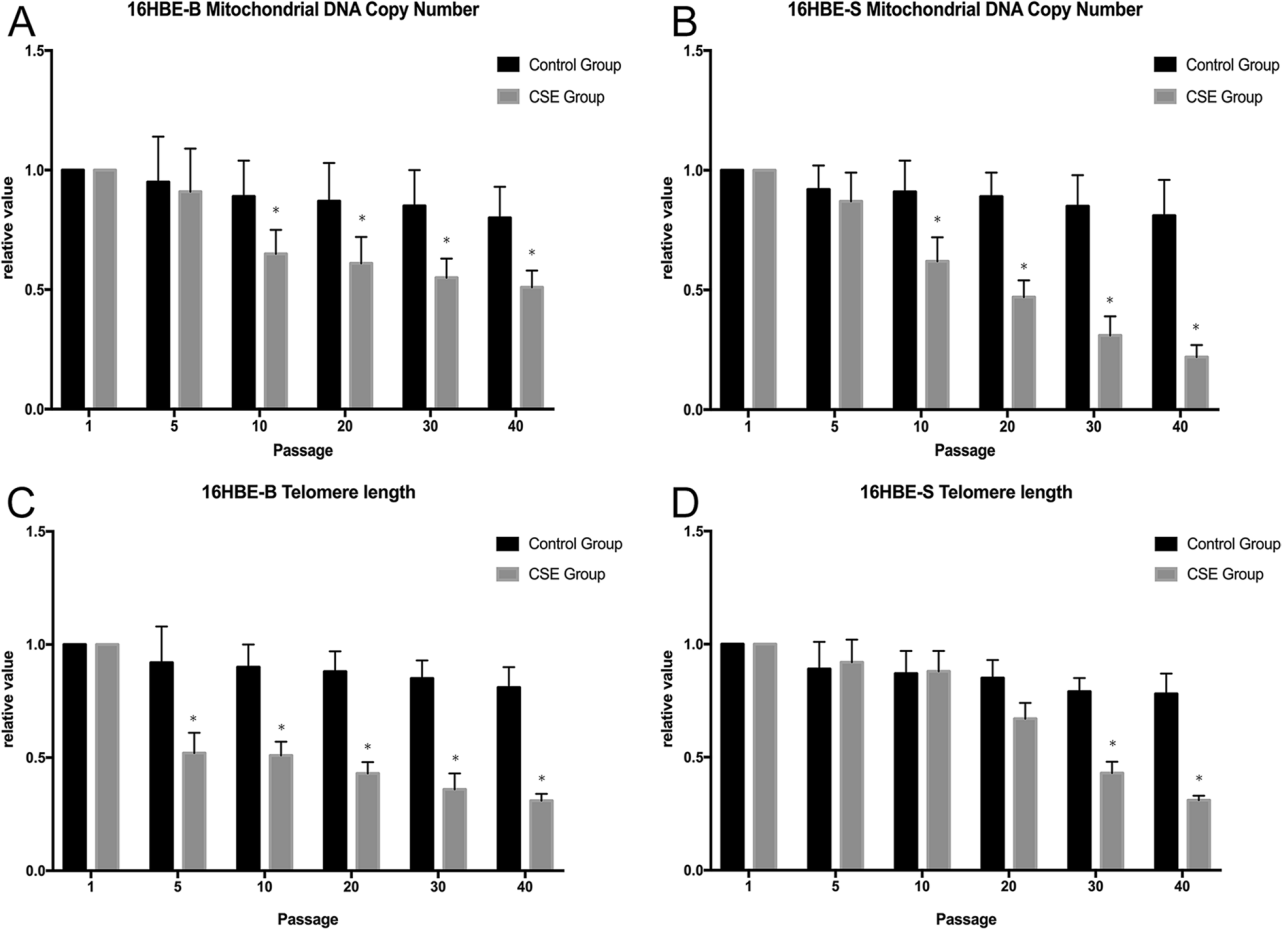
16HBE-B and 16HBE-S cell relative telomere length and mitochondrial DNA copy number changes across generations. **A** Mitochondrial DNA copy number for each generation of 16HBE-B cells. **B** Mitochondrial DNA copy number for each generation of 16HBE-S cells. **C** Relative telomere length change value for 16HBE-B in each generation. **D** Relative telomere length change value for 16HBE-S in each generation. *P ≤ *0.05*, n = 3

Through a qPCR experiment (as shown in Fig. 6), we detected each generation of 16HBE-B and 16HBE-S cells relative telomere length (Additional file 5, Additional file 6) and mitochondrial DNA (mtDNA) copy number (Additional file 7, Additional file 8).

As shown in Fig. 6A, B, compared with the control group, the mitochondrial DNA copy of 16HBE-B and 16HBE-S cell counts in the exposed group decreased from the 10th generation onward. Results were statistically significant.

The results of the relative telomere length detection experiment are shown in Figs. 6C–D. The results show that when compared with the control group, the cells in the 16HBE-B exposed group’s relative telomere length decreased from the 5th generation onward with statistical significance. The 16HBE-S exposed group’s relative telomere length decreased from the 20th generation onward, and this difference was statistically significant between the 30th and 40th generations.

### Chronic CSE exposure induces malignant transformation of different sizes of 16HBE

We tested the colony forming rate for each group of cells in double-soft agar (as shown in Table 6). The results showed that the DMSO control group and the CSE-treated group of 16HBE-B cells and 16HBE-S cells both failed to form obvious clones in the first to the tenth generations. However, the colony-forming rate increased from the 20th generation onward. Compared with the control group, the colony forming rate in each group in the 40th generation exceeded that of the 10th generation. The highest cell formation rates were 12% in the 16HBE-B group and 6% in the 16HBE-S group. All control groups’ clone formation rates were less than or equal to 1% induced by DMSO. These results showed that with the increase in CSE exposure and the passage of time, clone formation rate increased gradually. Compared with the control group, the CSE exposure group had obvious malignant transformation. Moreover, the differences between the 16HBE-B cells and the 16HBE-S cells were statistically significant.

**Table 6:** The clonal formation rate of different generations in 16HBE-S and 16HBE-B control group and the exposed group cell

Group	Clonal formation rate (%)					
Passage	1	5	10	20	30	40
16HBE-B control	0.01	0.01	0.01	0.01	0.03	1.00
16HBE-B CSE	0.01	0.01	0.01	0.05	3.00	12.0
16HBE-S control	0.00	0.00	0.00	0.01	0.07	0.70
16HBE-S CSE	0.00	0.00	0.01	0.05	0.40	6.00

**Fig. 7:**
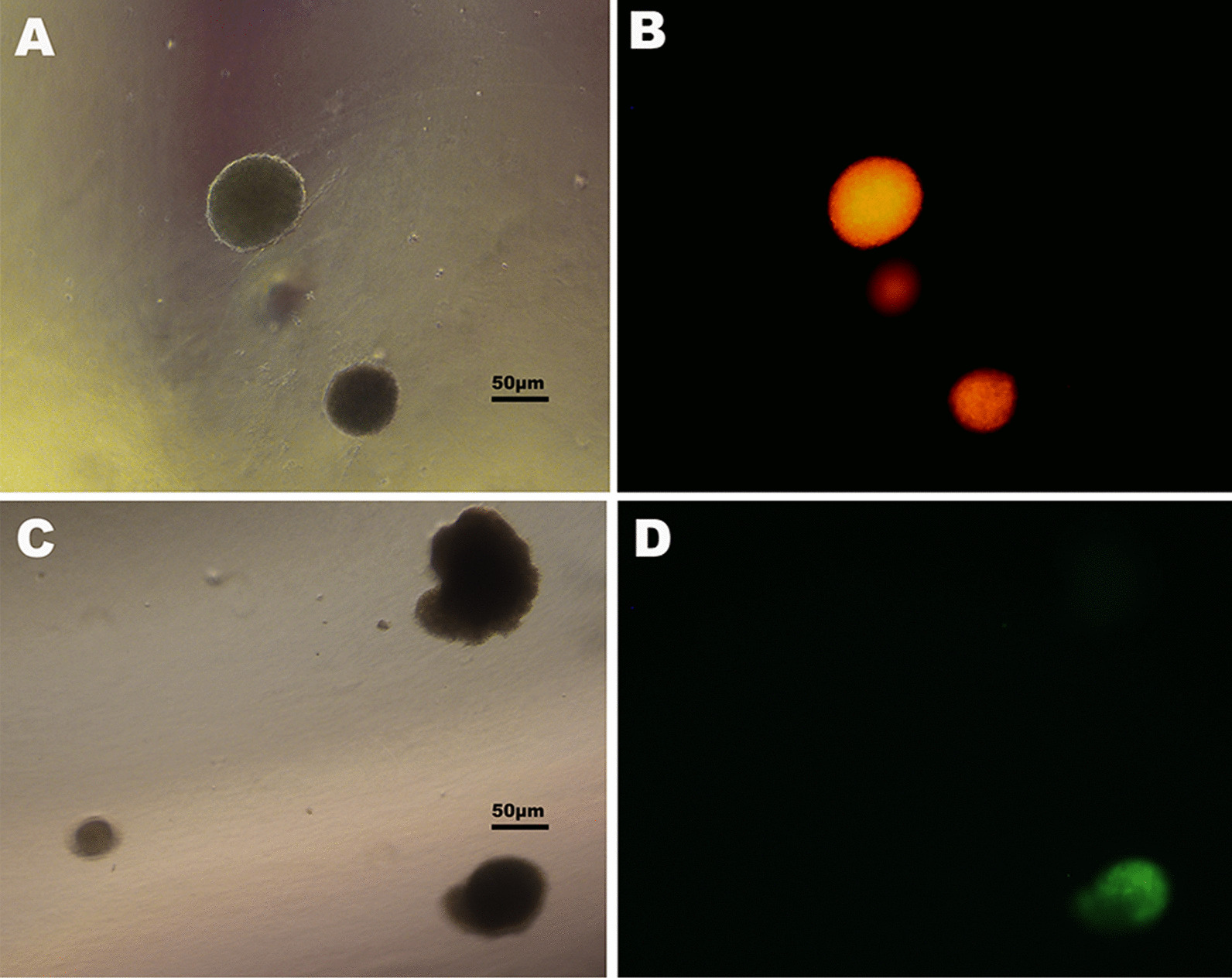
Colony formation in the 16HBE-B and 16HBE-S cells. **A** 16HBE-B colony formation in double soft agar. **B** 16HBE-B red fluorescent cloning in double soft agar. **C** 16HBE-S colony formation in double soft agar. **D** 16HBE-S green fluorescent cloning in double soft agar

Under a microscope, the CSE-treated cells formed cell mounds (Fig. 7), indicating a loss of contact inhibition. This was the first indication of malignant transformation.

As shown in Table 7, in the 40th passage, red fluorescent clones accounted for 0.79% of the total cells in the 16HBE-B control group and 11.4% in the 16HBE-B CSE exposure group. Green fluorescent clones accounted for 0% of the total cells in the 16HBE-S control group and 0.06% in the 16HBE-S CSE exposure group. The increase in red fluorescent clones in the 16HBE-B group and the disappearance of green fluorescent clones in the 16HBE-S group shows that the 16HBE-B cells are more likely to transform into cancer cells.

**Table 7:** The clonal formation rates of different generations in 16HBE-S and 16HBE-B control group and exposed group’s cell fluorescence

Group	Percentage of fluorescent clones (%)	
Passage	1	40
16HBE-B control	0	0.79
16HBE-B CSE	0	11.4
16HBE-S control	0	0
16HBE-S CSE	0	0.06

### Invasions of CSE-transformed cells

**Fig. 8:**
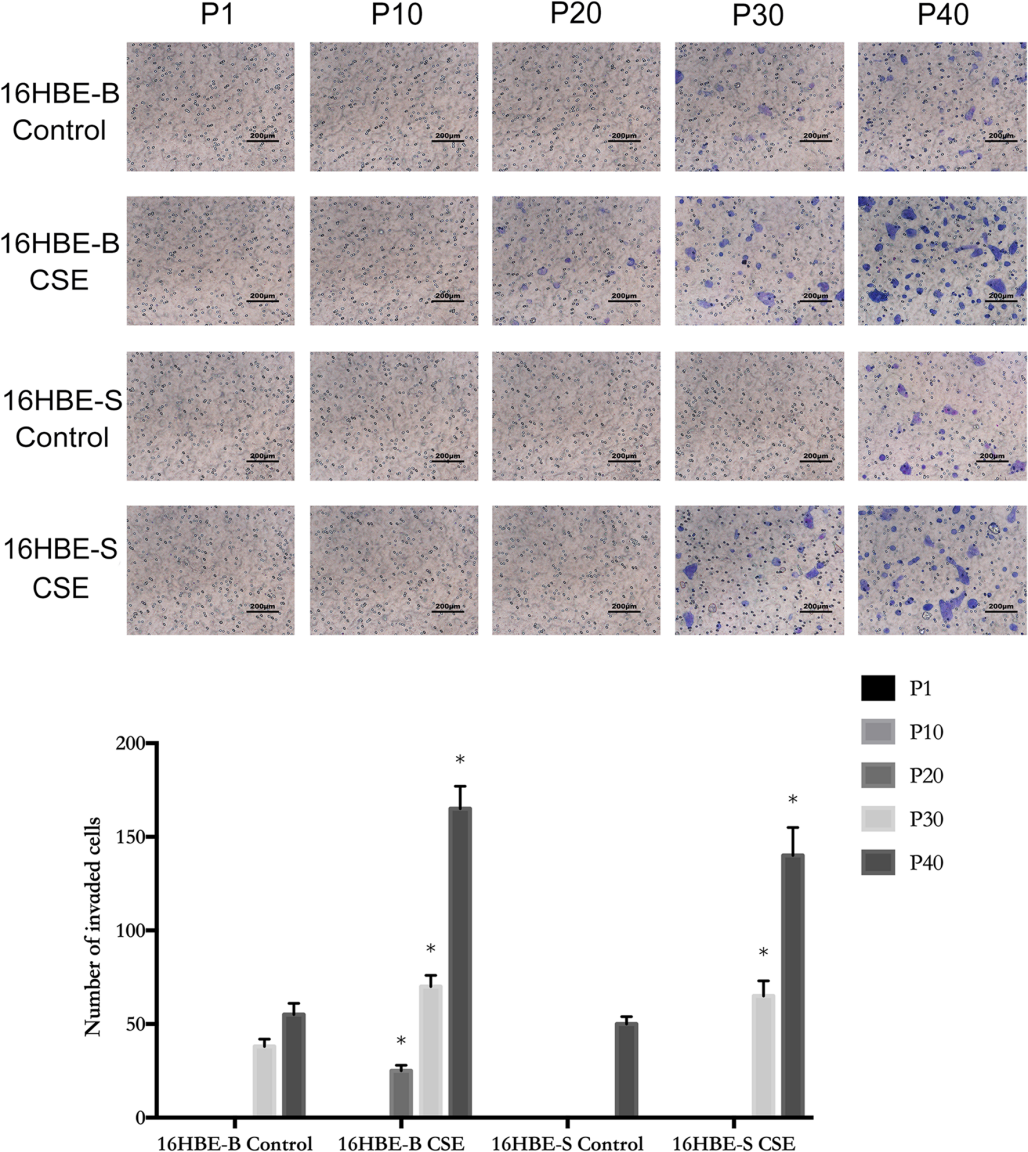
Transwell invasion assay and statistical analysis of different generations of the 16HBE-B and 16HBE-S group. *P ≤ *0.05*, n = 3, 16HBE-B CSE versus 16HBE-B Control, 16HBE-S CSE versus 16HBE-S Control

As shown in Fig. 8, the invasiveness of 16HBE-B cells gradually increased as the generations increased, and the invading cells in the exposure group appeared from the 20th generation to the 40th generation. Invading cells in the same generation of the control group increased as well, and these findings were statistically significant. 16HBE-S cell invasiveness gradually increased along with generational increase, in which the more obvious invading cells appeared from the 30th generation to the 40th generation in the exposed group. This growth difference was statistically significant when compared with the invading cell counts in the same generation of the control group.

### The stemness genes associated with CSE-transformed cells

We selected lung development and lung squamous cell carcinoma-related stem genes and related molecules of the epithelial-mesenchymal transition pathway for detection according to previous studies on the malignant transformation of CSE cells in phenotype change and differentiation. KRT5 and KRT14 are markers of basal cells. In the mouse lung squamous cell carcinoma model, the protein in the lung squamous cell carcinoma cells can strongly express KRT5/14. Some scholars speculate that the malignant transformation of basal cells may cause lung squamous cell cancer [21]. Another study proposes a human lung putative stem cell population, having multi-tissue differentiation potential, which can differentiate lung epithelial cells, mesenchymal and endothelial tissue, and the expression of pluripotency markers OCT4, NANOG, SOX2, and KLF4b [22]. Programmed death ligand-1 (PD-L1) is an immunomodulatory molecule that is involved in the tumor cell escape mechanism discovered in recent years [23, 24]. It is strongly expressed in tumor cells and interacts with the programmed death receptors (PD-1) in lymphocytes. The receptor (PD-1) binds to inhibit the lymphocytes’ immune effect on tumor cells [25]. Therefore, we selected the SOX2, SOX4, SOX9, KRT5, KRT14, NANOG and PD-L1 genes as our research objects (Additional file 9).

**Fig. 9:**
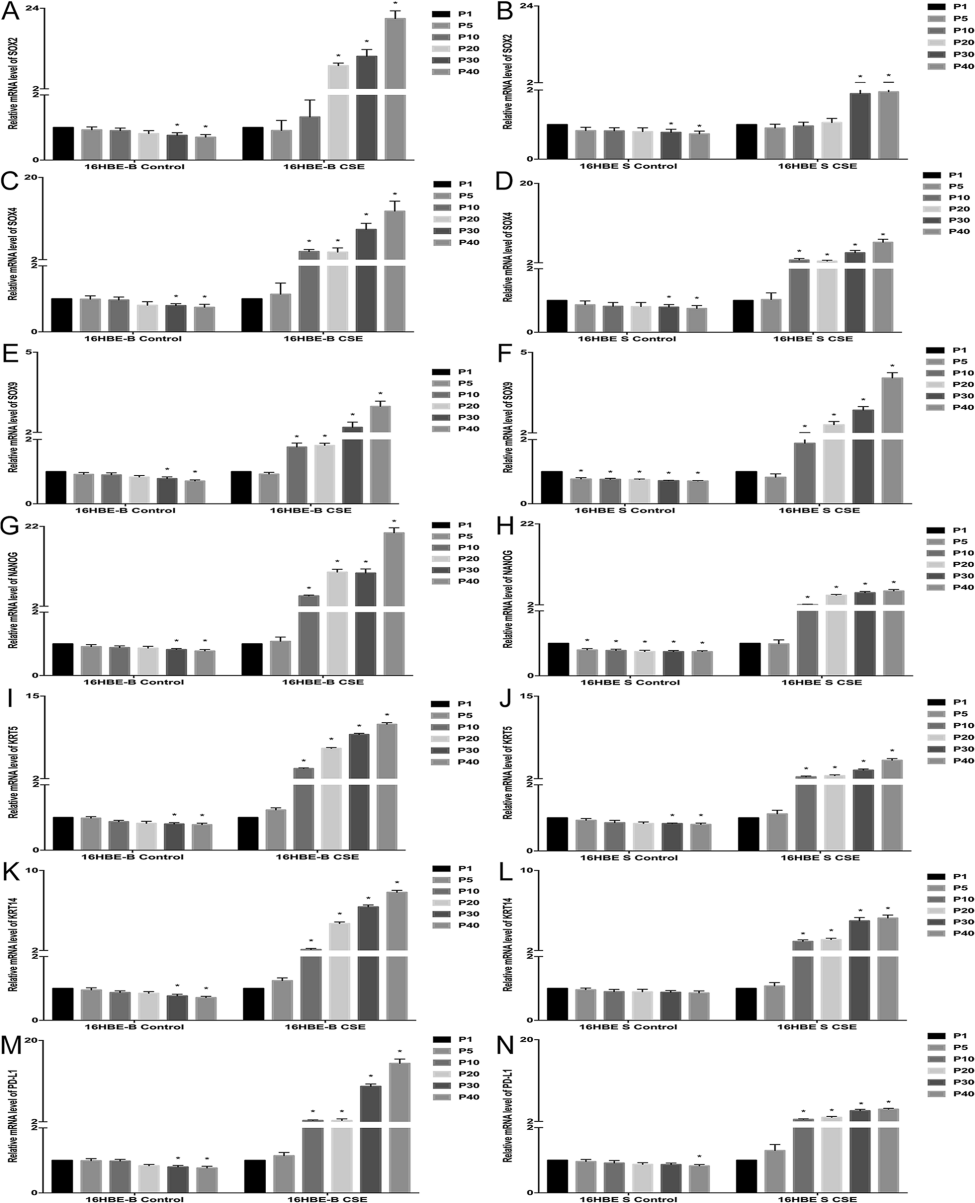
The stemness gene expression levels of the 16HBE-B and 16HBE-S cells of different generations. **A** The relative mRNA level of SOX4 expression in the 16HBE-B control and CSE groups. **B** The relative mRNA level of SOX4 expression in the 16HBE-S control and CSE groups. **C** The relative mRNA level of SOX2 expression in the 16HBE-B control and CSE groups. **D** The relative mRNA level of SOX2 expression in the 16HBE-S control and CSE groups. **E** The relative mRNA level of SOX9 expression in the 16HBE-B control and CSE groups. **F** The relative mRNA level of SOX9 expression in the 16HBE-S control and CSE groups. **G** The relative mRNA level of NANOG expression in the 16HBE-B control and CSE groups. **H** The relative mRNA level of NANOG expression in the 16HBE-S control and CSE groups. **I** The relative mRNA level of KRT5 expression in the 16HBE-B control and CSE groups. **J** The relative mRNA level of KRT5 expression in the 16HBE-S control and CSE groups. **K** The relative mRNA level of KRT14 expression in the 16HBE-B control and CSE groups. **L** The relative mRNA level of KRT14 expression in the 16HBE-S control and CSE groups. **M** The relative mRNA level of PD-L1 expression in the 16HBE-B control and CSE groups. **N** The relative mRNA level of PD-L1 expression in the 16HBE-S control and CSE groups. *P ≤ *0.05, n* = *3,* **P ≤ *0.01, n* = *3, *versus* P1* Group

As shown in Fig. 9A–B, the SOX4 gene’s expression in the 16HBE-B, 16HBE-S and cell control groups decreased as the generation increased. Compared to the first generation of control group cells in each group, the difference between 16HBE-B and 16HBE-S cells from the 30th representative onward was statistically significant. The SOX4 gene’s expression in 16HBE-B and 16HBE-S cells increased along with the increase in cellular generation. Compared with the cells in the first generation, the difference in expression and growth of 16HBE-B and 16HBE-S from the 10th generation onward was statistically significant.

As shown in Fig. 9C–D, compared with the first generation of control group cells in each group, the SOX2 gene’s expression in the 16HBE-B and 16HBE-S cells decreased from the 30th generation to the 40th generation, and this was statistically significant. The SOX2 gene’s expression in the 16HBE-B and 16HBE-S cells also increased with the increase in cellular generation. Compared to the first generation of cells, SOX2’s expression in 16HBE-B increased beginning in the 20th generation, and SOX2’s expression in 16HBE-S increased beginning in the 30th generation. Each of these results had statistical significance.

As shown in Fig. 9E–F, the SOX9 gene’s expression in the 16HBE-B and 16HBE-S cell control groups trended downward as cellular generations increased. Compared with the first generation of control group cells in each group, the difference in 16HBE-S cells from the 5th representative downward was statistically significant, and the difference in 16HBE-B cells from the 30th generation onward was statistically significant. The SOX9 gene’s expression in the 16HBE-B and 16HBE-S cells increased along with the increase in cellular generation. Compared with the cells in the first generation, the expression of 16HBE-B and 16HBE-S in the 10th generation increased, also with statistical significance.

As shown in Fig. 9G–H, the NANOG gene’s expression in the 16HBE-B and 16HBE-S cell control groups trended downward with the increase in cellular generation. Compared with the first generation of control cells in each group, the 16HBE-S cells’ expression decreased from the fifth generation onward, and the 16HBE-B cells’ expression decreased from the 30th generation onward. However, the NANOG gene’s expression in the 16HBE-B and 16HBE-S cells increased with the increase in cellular generation. Compared with the cells in the first generation of each group, the expression of 16HBE-B and 16HBE-S began increasing in the 10th generation.

As shown in Fig. 9I–J, the KRT5 gene’s expression in the 16HBE-B and 16HBE-S cell control groups trended downward with the increase in cellular generation. Compared with the first generation of control cells in each group, the decline in the 16HBE-B and 16HBE-S cells from the 30th generation onward was statistically significant. In addition, the KRT5 gene’s expression in the 16HBE-B and 16HBE-S cells increased with the increase in cellular generation. Compared with the cells in the first generation, the expression of 16HBE-B and 16HBE-S in the 10th generation increased, and this was statistically significant.

As shown in Fig. 9K–L, the KRT14 gene’s expression in the 16HBE-B and 16HBE-S cell control groups trended downward as cellular generation increased. Compared with the first generation of control group cells in each group, the decline in 16HBE-B cells from the 30th generation onward was statistically significant. Although the 16HBE-S cells’ expression appeared to trend downward, the difference was not statistically significant. The KRT14 gene’s expression in 16HBE-B and 16HBE-S cells increased with the increase in cellular generation. Compared with cells in the first generation, the expression of 16HBE-B and 16HBE-S in the 10th generation increased, with statistical significance.

As shown in Fig. 9M–N, the PD-L1 gene’s expression in the 16HBE-B and 16HBE-S cell control groups trended downward with the increase in cellular generation. Compared with the first generation of control group cells in each group, the decline in 16HBE-B cells from the 30th representative onward was statistically significant, and that of the 16HBE-S cells from the 40th generation onward was statistically significant as well. The PD-L1 gene’s expression in the 16HBE-B and 16HBE-S cells increased with the increase in cellular generation. Compared with the cells in the first generation, the expression of 16HBE-B and 16HBE-S in the 10th generation increased, with statistical significance.

Finally, we compared 16HBE-B and 16HBE-S cells’ gene expression levels. There was no significant difference between the 16HBE-B and 16HBE-S cell control groups, but the gene expression in the 16HBE-S control group decreased, and this was statistically significant. The gene expression of SOX9 and NANOG decreased from the 5th generation onward with statistical significance. However, the 16HBE-S CSE treatment group’s stemness gene expression levels were lower than those of the 16HBE-B CSE treatment group.

### E-cadherin mediates epithelial mesenchymal transformation in different sizes of CSE-transformed 16HBE cells

**Fig. 10:**
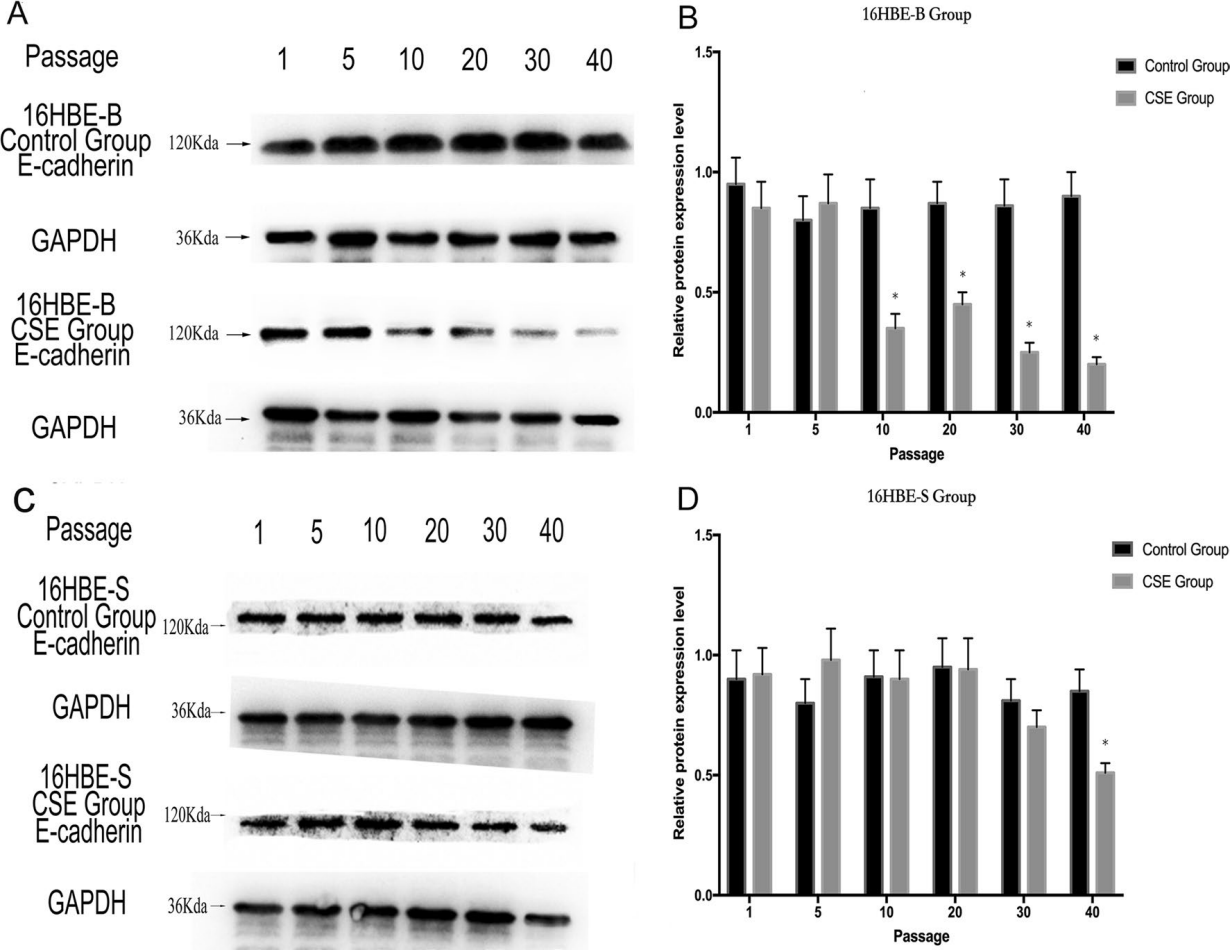
E-cadherin expression in the 16HBE-B and 16HBE-S cells. **A** E-cadherin expression in the 16HBE-B cells of different generations. **B** E-cadherin expression in the 16HBE-B cells of different generations. **C** E-cadherin expression in the 16HBE-S cells of different generations. **D** E-cadherin expression in the 16HBE-S cells of different generations. *P ≤ *0.05*, n = 3, 16HBE-B CSE versus 16HBE-B Control, 16HBE-S CSE versus 16HBE-S Control

We used Western blot to detect E-cadherin protein expression in the 16HBE-B and 16HBE-S cells (as shown in Fig. 10). The results showed that E-cadherin protein decreased with the increase of cell numbers in each group (Additional file 10, Additional file 11). Compared with the control group, the decrease in E-cadherin protein expression in the 16HBE-B group was statistically significant from the C10 generation onward. In addition, the decrease in E-cadherin protein expression in the C40 generation in the 16HBE-S group was statistically significant, but vimentin protein could not be detected.

### The relationship between cell level and squamous lung cell carcinoma pathogenesis

**Fig. 11:**
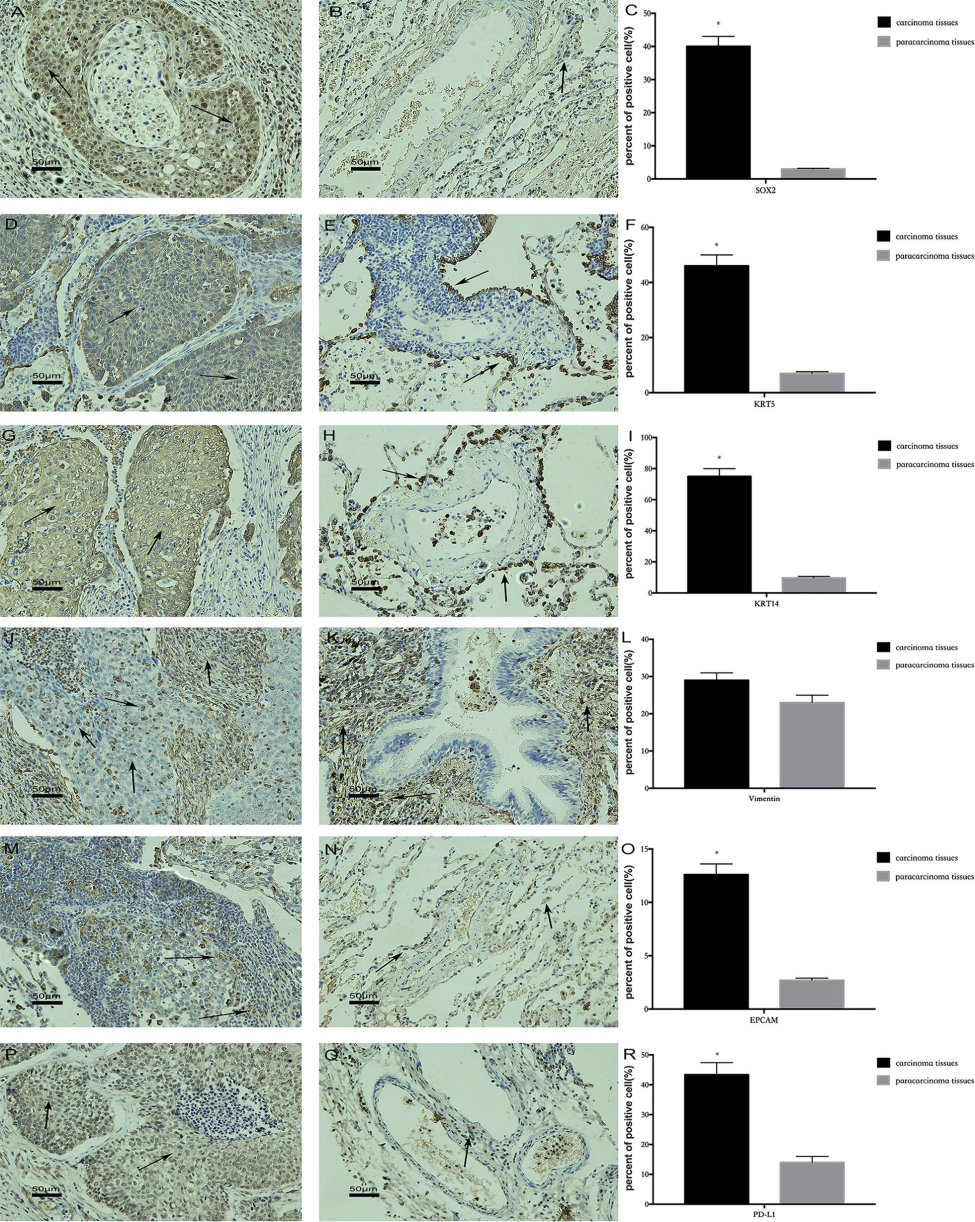
Stemness protein expression in lung squamous cell carcinoma and adjacent tissues. **A** SOX2 expression in cancer. **B** SOX2 expression in adjacent tissues. **C** SOX2 expression in cancer and adjacent tissues. **D** KRT5 expression in cancer. **E** KRT5 expression in adjacent tissues. **F** KRT5 expression in cancer and adjacent tissues. **G** KRT14 expression in cancer. **H** KRT14 expression in adjacent tissues. **I** KRT14 expression in cancer and adjacent tissues. **J** Vimentin expression in cancer. **K** Vimentin expression in adjacent tissues. **L** Vimentin expression in cancer and adjacent tissues. **M** EPCAM expression in cancer. **N** EPCAM expression in adjacent tissues. **O** EPCAM expression in cancer and adjacent tissues. **P** PD-L1 expression in cancer. **Q** PD-L1 expression in adjacent tissues. **R** PD-L1 expression in cancer and adjacent tissues. *P ≤ *0.05*, n = 3, cancer adjacent tissues versus adjacent tissues

Previous cell experiments have shown that in the process of CSE-induced malignant 16HBE cell transformation, the expression of stemness genes in larger cells rises, and they dedifferentiate through the epithelial mesenchymal transformation pathway to generate malignant transformation. Therefore, we selected SOX2, KRT5 and KRT14 stemness gene proteins, PD-L1 immune related proteins, vimentin protein, and EpCAM protein, all of which influence epithelial carcinogenesis, to explore whether the mechanism at the cell level corresponds to lung squamous cell carcinoma pathogenesis (Fig. 11). We compared these six proteins’ expression in and around the lung cancer. The immunohistochemistry results showed high expressions of KRT5, KRT14, SOX2, PD-L1 and EpCAM in the lung squamous cell carcinoma (Fig. 11C/F/I/L/O/R). Additionally, we found vimentin in the adjacent mesenchymal tissue, but not in the epithelial cells, while a small amount of vimentin was detected in the epithelial cells.

## Discussion

Lung squamous cell carcinoma is a common malignancy, which affects roughly 400,000 patients each year worldwide, and the vast majority of lung squamous cell carcinoma patients are heavy smokers [26, 27]. There is a 95% correlation between lung squamous cell carcinoma and smoking. Cancer Genome Atlas (TCGA) research has shown that lung squamous cell carcinoma of the genome is more complex, and the tobacco carcinogenesis has a high mutation load [28].

The previous view was that the basal cells involved in airway epithelial repair may be the initial cells of lung squamous cell carcinoma [29]. Some basal cells have the characteristics of stem cells, primarily in the G0 phase of the cell cycle in a resting state, with long cell life and unlimited proliferation. Therefore, cigarette smoke causes basal cell gene mutations and accumulates to transform into lung squamous cell carcinoma stem cells, which then generate lung squamous cells. Barth et al. showed that in the existing mouse lung squamous cell carcinoma model, lung squamous cell carcinoma can strongly express KRT5/14 protein, a specific surface marker of basal cells [21]. Some researchers have contended that the malignant transformation of cells may lead to lung squamous cell carcinoma [30–33]. Debates persist over whether lung squamous cell carcinoma is derived from rapidly dividing but abnormal stem cells, or from differentiated cells that produce precancerous lesions with all the accumulated mutations returning to a stem cell-like state to generate squamous cell carcinoma. There are many types of epithelial cells in the body’s bronchial epithelial cells which have a degree of heterogeneity [12, 34]. Therefore, we should not only consider the heterogeneity of lung squamous cell carcinoma cells, but we should also consider the role of normal bronchial epithelial cell heterogeneity in the process of malignant transformation.

The past few decades have witnessed great strides in clarifying the original cells of various malignancies [35]. However, there has only been scant research on susceptible cells and initiating cells using the heterogeneity of normal bronchial epithelial cells through malignant transformation. Therefore, we sorted cells by size to research 16HBE’s heterogeneity. Because heterogeneity originates in the embryonic stage of lung development, we also needed to use the lung’s key developmental genes during the embryonic stage to study heterogeneity’s role in malignant transformation.

Lung bronchial epithelium heterogeneity originates in the embryonic period. In the tubule stage of lung development, SOX gene families, including SOX2, SOX4, and SOX9, are widely expressed in the developing lung. The SOX gene is also associated with lung cell heterogeneity which can act as an oncogene in the occurrence and progression of tumors [36], affect their genesis, differentiation, metastasis and prognosis [37–41].

Therefore, we used a stemness gene associated with tumor cell heterogeneity and SOX2, SOX4, SOX9, basal cell markers KRT5, KRT14, immune genes PD-Ll and epithelial-mesenchymal transition E-cadherin to study heterogeneous cell and malignant transformation mechanisms. Then, we related the molecules with human lung squamous cell carcinoma expression for mutual confirmation. Through these studies, we validated 16HBE heterogeneity and the various phenotypic and molecular expression in malignant transformation induced by CSE.

The low-dose CSE-induced in vitro cell malignant transformation experiment is suitable for simulating cigarettes’ malignant transformation effect on bronchial cells. Cell heterogeneity sorting is based on cell size. The cell sorting diagram shows that the 16HBE-B cell group is the main cell group, presenting an obvious aggregation state, while the 16HBE-S cell group is scattered and there is no obvious cell grouping. In addition to the cells, the 16HBE-S group also contains cell debris.

The cell fluorescence results show an immediately obvious difference. With exposure to CSE and passage, 16HBE-B’s red proportion of fluorescent cells gradually increased, while 16HBE-S’s proportion of green fluorescent cells gradually decreased. We think that the increased proportion of red fluorescent cells was due to the enhanced malignant transformation and proliferation ability of the cells with red fluorescence in the 16HBE-B group. Meanwhile, the decreased proportion of green fluorescent cells was maybe due to the enhanced malignant transformation and proliferation ability of the cells without green fluorescence in the 16HBE-S group. This result may be explained by the fact that the stem cells in the 16HBE-S group were maybe not actually transfected with green fluorescent genes. However, the newly differentiated stem cells are more likely to be affected by CSE in the division phase due to their strong proliferation ability. Moreover, the proportion of green fluorescent cells decreases due to the proliferation of non-fluorescent new cells. In the 16HBE-B group, there are less stem cells, so there are also less new non-fluorescent cells, and finally, the proportion of red fluorescent cells increased. This is consistent with the results of the soft agar cloning experiment, but which need more experimental results to verify.

The cell phenotype changes in the cigarette-induced malignant cell transformation process, as well as the susceptible cells’ heterogeneity, are the problems we sought to resolve. We used cell morphology, growth curve, cell cycle, cell fluorescence, mitochondrial relative telomere length and DNA copy numbers to evaluate cell malignant transformation and the difference in phenotypic changes. We used soft agar cloning and nude mouse tumor formation to observe the degree of malignant transformation, and to explore the relationship between cell heterogeneity and malignant transformation. This lays a foundation for further research on the mechanism of malignant transformation in CSE cells.

We found that CSE-induced 16HBE series malignantly transformed cells alter cell morphology: cell proliferation increases, the cell cycle’s G1 phase shortens, and relative telomere length and mitochondrial DNA copy number decline. The changes in relative telomere length and mitochondrial DNA copy number among the 16HBE-B and 16HBE-S group show heterogeneity between them, and that large cell size is susceptible to malignant transformation induced by CSE. In an American national health and nutrition examination survey about associations of smoking indicators with telomere length shows the increased number of cigarette consumption to be associated with shorter telomere length in both Blacks and Whites, indicating that the impact of smoking on life-shortening diseases could partly be explained by telomere biology [42]. Another study showed a modification effect of the postconceptual age, indicating that older fetuses with prenatal smoking exposure had shorter telomere length than their counterparts [43]. At present, mitochondrial DNA copy number changes have been detected in many tumors, such as liver cancer, prostate cancer, breast cancer and colorectal cancer [44–47]. In human breast epithelial cell MCF10A, the copy number of mitochondrial DNA reduction can induce epithelial-mesenchymal transformation and produce the breast cancer stem cells [48]. In prostatic epithelial cells PNT1A, the reduction of mitochondrial DNA copy number prevents cell apoptosis and promotes cell invasion and metastasis by activating PI3K/AKT2 signalling pathway, which further explains the reduction of mitochondrial DNA copy number can lead to malignant transformation of epithelial cells [49]. Meanwhile, some studies have found that reduction of the copy number of mitochondrial DNA in breast cancer and prostate cancer cells can promote the increase of the malignant degree of tumor cells [48, 50]. The above fully explained the important role of mitochondrial DNA copy number changes in cell phenotypes and tumorigenesis development.

In our study, chronic exposure of CSE resulted in shorter telomeres and mitochondrial DNA copy number decline in 16HBE cells. So, smoking will accelerate telomere attrition in humans. The higher rate of shorter telomeres and the reduction of mitochondrial DNA copy number may mediate the link between smoking and malignant transformation to an increased risk of several lung-cancer diseases.

Epithelial cell adhesion molecule (EpCAM) is abnormally expressed in a variety of human tumor tissues and is involved in tumor genesis [51–53]. SOX2 inhibits the expression of CDKN1A (cell cycle inhibitor) and maintains the lung squamous cell tumor cell growth through this mechanism [54]. SOX2 can be utilized as the primary regulator of tumor squamous cell differentiation. Studies have shown that the SOX2 in tracheobronchial cells overexpressed with CDKN2A and PTEN deletion combination results in lung squamous cell carcinoma production [55]. PD-L1 protein expression can be detected in various human tumor tissues. PD-L1 expression was significantly upregulated in cancer tissues compared to the normal tissues [56].

The CSE-induced malignant transformation of 16HBE-B and 16HBE-S cells alters these cells at the molecular level. Several stemness and immunity gene expression levels show that compared with a control group, the SOX2, SOX4, SOX9, NANOG, KRT5, KRT14, and PD-L1 genes in 16HBE-B and 16HBE-S expression of malignant transformed cells showed a significant increase. Moreover, the 16HBE-B cells’ expression level changed more than that of the 16HBE-S cells. In the control group, each of these genes trended downward. The expression levels for the SOX9 and NANOG genes in the 16HBE-S group’s control group decreased, to an extent that was found to be statistically significant. After the cells underwent CSE malignant transformation, the cell stemness genes’ expression increased. In the control group, 16HBE-S cell stem gene expression decreased, and this was also statistically significant. This may have been due to stem cell differentiation. E-cadherin expression in the 16HBE exposure group decreased, especially in the 16HBE-B group. This is also reflected in previous results from soft agar cloning. However, a Western blot showed no vimentin presence, and it is likely that the protein content could not reach the detection limit due to the low number of transformed cells. The immunohistochemistry experiments on lung squamous cell carcinoma show that the lung tissues SOX2, KRT 5, KRT14, EPCAM and PD-L1 have a significant expression. Additionally, vimentin has minimal expression in cancer tissue, which explains the genesis and progression of squamous cell carcinoma of the lung when it is CSE-induced. This also explains why no vimentin bands were produced in the Western blot experiment.

## Conclusions

In summary, we believe that the mechanisms of CSE-induced 16HBE malignant transformation reduce 16HBE relative telomere length in larger size cells, causing stemness gene expression upregulation. The accumulated mass epithelial cells with larger size are transformed into cells of stemness ability through EMT, and these cells slowly form malignant tumors. It is likely that lung squamous cell carcinoma caused by smoking also operates through this mechanism. This is caused in part by CSE-induced 16HBE series cell malignant transformation and can simulate the genesis of human lung squamous cell carcinoma. This is a starting point for the full mechanism, but the results laid out in this paper provide an important reference for the etiology of lung squamous cell carcinoma. In this manuscript, we found that the ability of cigarette smoke extract to cause malignant transformation of the human larger size bronchial epithelial cells through EMT which are related with E-cadherin expression change. The findings in our work provide novel insights for understanding the impact of cellular heterogeneity in lung cancer development.

## Supplementary Information

**Additional file 1.** CSE growth inhibition rate on 16HBE-B and 16HBE-S.

**Additional file 2.** Growth curve date of 16HBE-B cells and 16HBE-S cells of different generations.

**Additional file 3.** Mitochondrial DNA copy number for each generation of 16HBE-B cells.

**Additional file 4.** Mitochondrial DNA copy number for each generation of 16HBE-S cells.

**Additional file 5.** Relative telomere length change value for 16HBE-B in each generation.

**Additional file 6.** Relative telomere length change value for 16HBE-S in each generation.

**Additional file 7.** The stemness gene expression levels of 16HBE-B and 16HBE-S cells of different generations.

**Additional file 8.** Original Image for checking-16HBE-B CSE and Control group.

**Additional file 9.** Original Image for checking-16HBE-S CSE and Control group.

**Additional file 10.** Certificate of STR Analysis of 16HBE(HBE135-E6E7)ENCN and Transfer agreement.

**Additional file 11.** Detection of Mycoplasmas.

## Data Availability

All data generated or analyzed during this study are included in this published article and its supplementary information files.

## Abbreviations

CSE: Cigarette smoke extract
16HBE: Human bronchial epithelial cells
EMT: Epithelial-mesenchymal transition
IARC: International agency for research on cancer
NNK: Methylnitrosaminepyridinone
FSC: Forward scattered light
LSC: Laterally scattered light
PD-L1: Programmed death ligand-1
MET: Mesenchymal-epithelial transition
PBS: Phosphate-buffered saline
PAHs: Polycyclic aromatic hydrocarbons
