# Supplementary material for: Strategies for understanding the role of cellular heterogeneity in the pathogenesis of lung cancer: a cell model for chronic exposure to cigarette smoke extract
Source: BMC Pulm Med. 2022 Sep 2;22:333. doi: 10.1186/s12890-022-02116-6 (PMC9438261; doi:10.1186/s12890-022-02116-6)
Supplement: Supplementary file 1 — Additional file 1. CSE growth inhibition rate on 16HBE-B and 16HBE-S. [file 12890_2022_2116_MOESM1_ESM.pdf]

Cytogenetic quality identification test report

Item name: Cell line

Test type: STR genotype test

Sample No.:

Table 1, Sample No

Customer Sample Number	Company number
355	20170925-06

Number of samples, quantity: 1

Sample traits: cell line

Test item: STR

Detection method: The DNA was extracted with the Axygen genomic extraction kit and amplified by the 20STR amplification protocol in the type ABI 3730XL heritage

The STR locus and the sex gene Amelogenin were tested on the transfer analyzer.

result:

(1) Inspection of the basic information

Table 2: Sample genotype test results

	multiple alleles	Matching cell lines	cell bank	EV price	Matching instructions
20170925-06	not have	HBE 135-E 6E 7	A TCC	1.00	Complete match

●Multiallelic gene refers to the triallelic and above gene phenomenon.

●The typing result of each cell was good.

(2) Description of each sample

●20170925-06: Cell DNA typing of this strain found the fully matched cell line in the cell line search, and the ATCC database shows the cell name as

HBE 135-E 6E 7 , The cell number corresponds to CRL-2741_. This assay did not identify multialleles in this cell line.

(3) Sample subtyping results

Genotyping results for the STR and Amelogenin loci of the cell 20170925-06						
Loci	Cell STR information was sent			Cell bank-cell STR information		
	Cell name was submitted for inspection: 355			Cell library cell name: HBE 135-E 6E 7		
	Allele 1	Allele 2	Allele 3	Allele 1	Allele 2	Allele 3
D 5S 818	11	12		11	12	
D 13S 317	8	11		8	11	
D 7S 820	9	10		9	10	
D 16S 539	9	12		9	12	
VW A	16	17		16	17	
TH 01	5	6		5	6	
AMEL	X	Y		X	Y	
T POX	8	10		8	10	
CSF 1PO	9	11		9	11	

D 12S 391	19	24				
FG A	24	25				
D 2S 1338	16	17				
D 21S 11	28	30				
D 18S 51	12	12				
D 8S 1179	13	14				
D 3S 1358	15	17				
D 6S 1043	13	13				
P E NTAE	12	16				
D 19S 433	13	16				
PENTA D	9	12				

Other instructions:

(I) Classification scheme and site distribution:

Schedule: Experimental protocol and site

	**Scheme 1**	**Scheme 2**	**Scheme 3**	**Scheme 4**
1	TH 01	T POX	D 3S 135 8	A MEL

2	D 12S 39 1	V WA	D 13S 31 7	D 5S 818
3	D 7S 820	D 8S 1179	D 6S 104 3	D 2S 1338
4	CSF 1PO	P ENTAD	D 16S 53 9	D 21S 11
5	F GA		D 19S 43 3	D 18S 51
6	PENTAE			

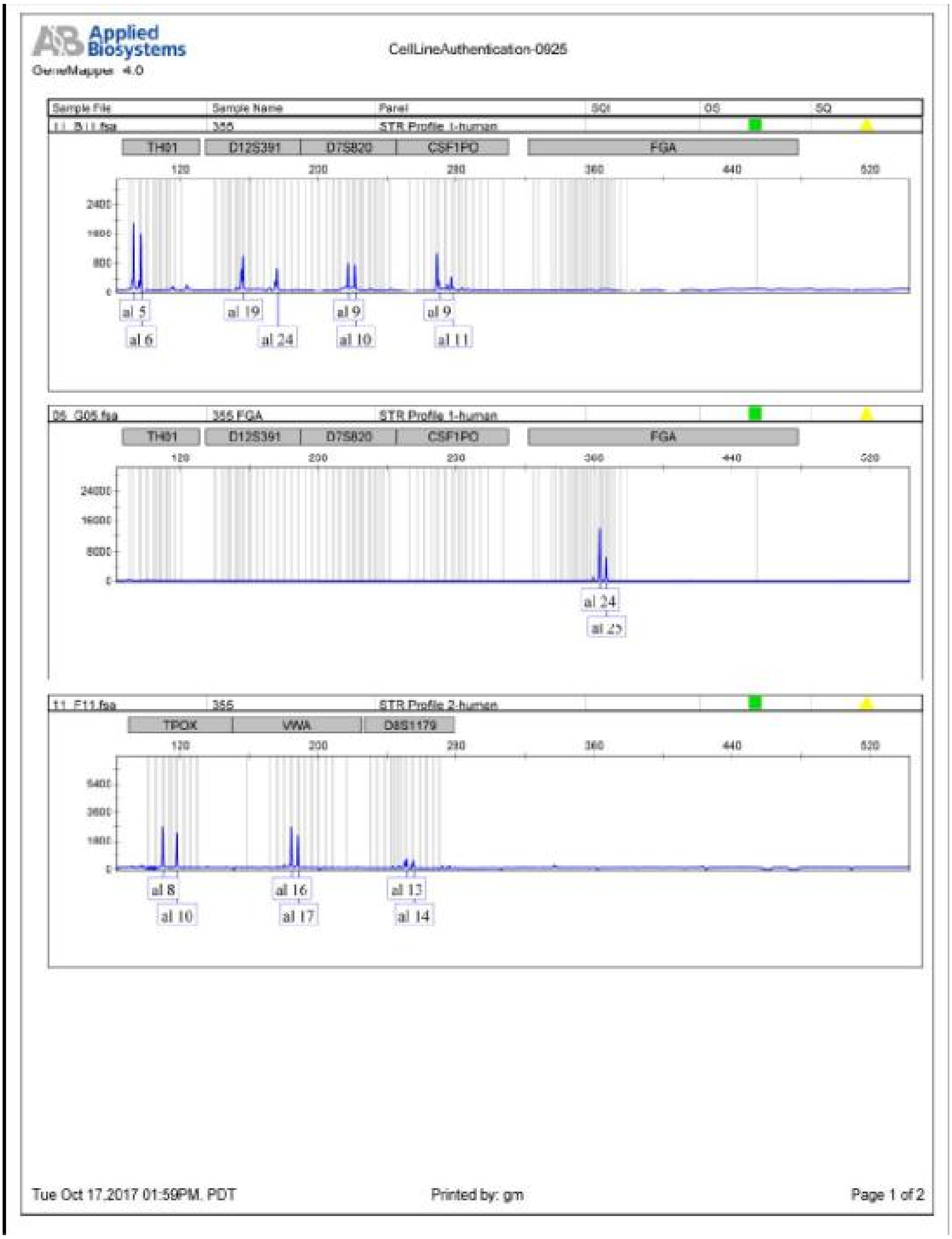
Applied Biosystems
GeneMapper 4.0
CellLineAuthentication-0925
Sample File | Sample Name | Panel | SQI | OS | SQ
11_B11.fsa | 355 | STR Profile 1-human
TH01 D12S391 D7S820 CSF1PO FGA
al 5 al 6 al 19 al 24 al 9 al 10 al 9 al 11
05_G05.fsa | 355 FGA | STR Profile 1-human
TH01 D12S391 D7S820 CSF1PO FGA
al 24 al 25
11_F11.fsa | 355 | STR Profile 2-human
TPOX VWA D8S1179
al 8 al 10 al 16 al 17 al 13 al 14
Tue Oct 17,2017 01:59PM, PDT
Printed by: gm
Page 1 of 2

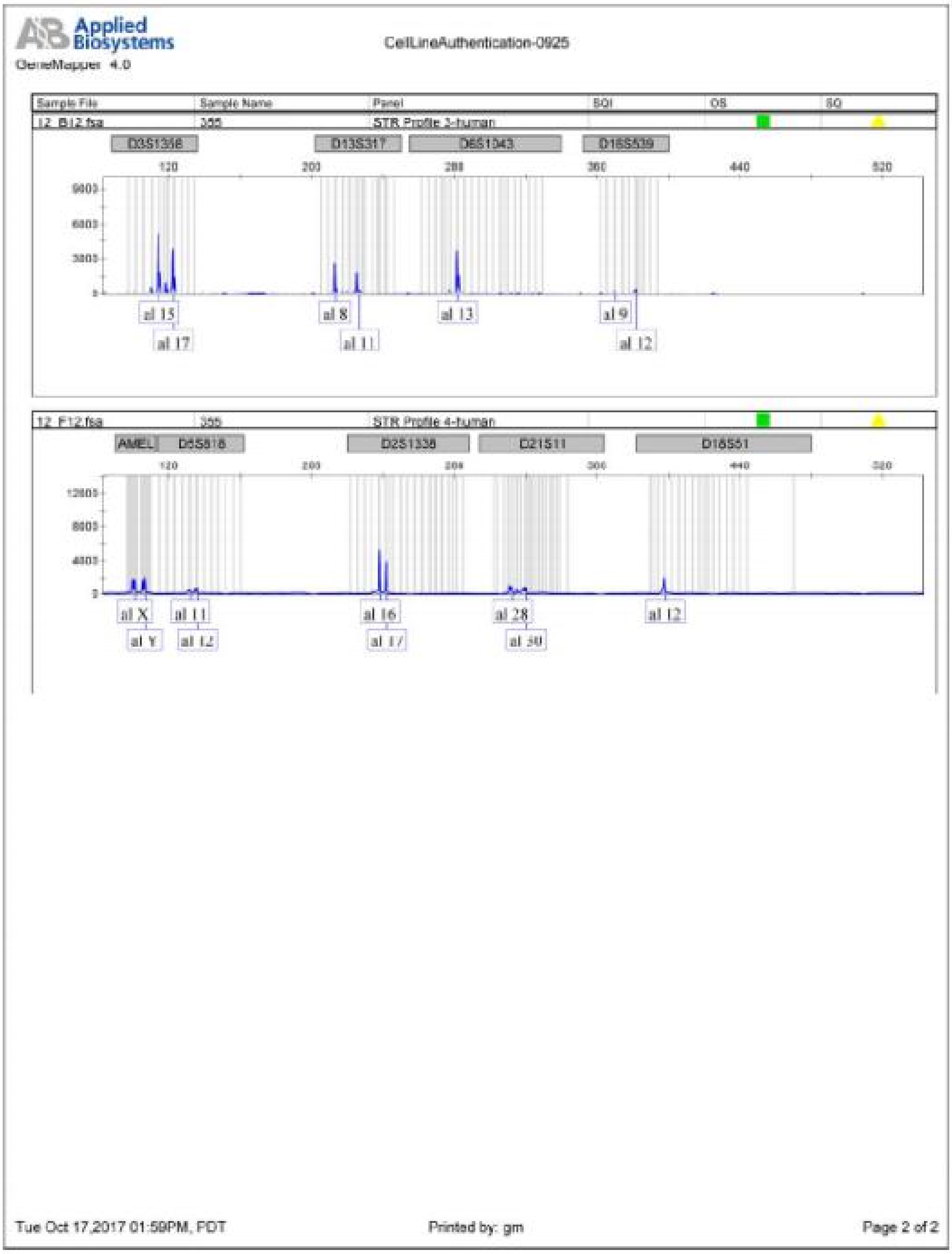
Applied Biosystems
GeneMapper 4.0
CellLineAuthentication-0925
Sample File
Sample Name
Panel
SQI
OS
SQ
12_B12.fsa
355
STR Profile 3-human
D3S1358
D13S317
D6S1043
D16S539
al 15
al 17
al 8
al 11
al 13
al 9
al 12
12_F12.fsa
355
STR Profile 4-human
AMEL
D5S818
D2S1338
D21S11
D18S51
al X
al Y
al 11
al 12
al 16
al 17
al 28
al 30
al 12
Tue Oct 17,2017 01:59PM, PDT
Printed by: gm
Page 2 of 2

Certificate of STR Analysis

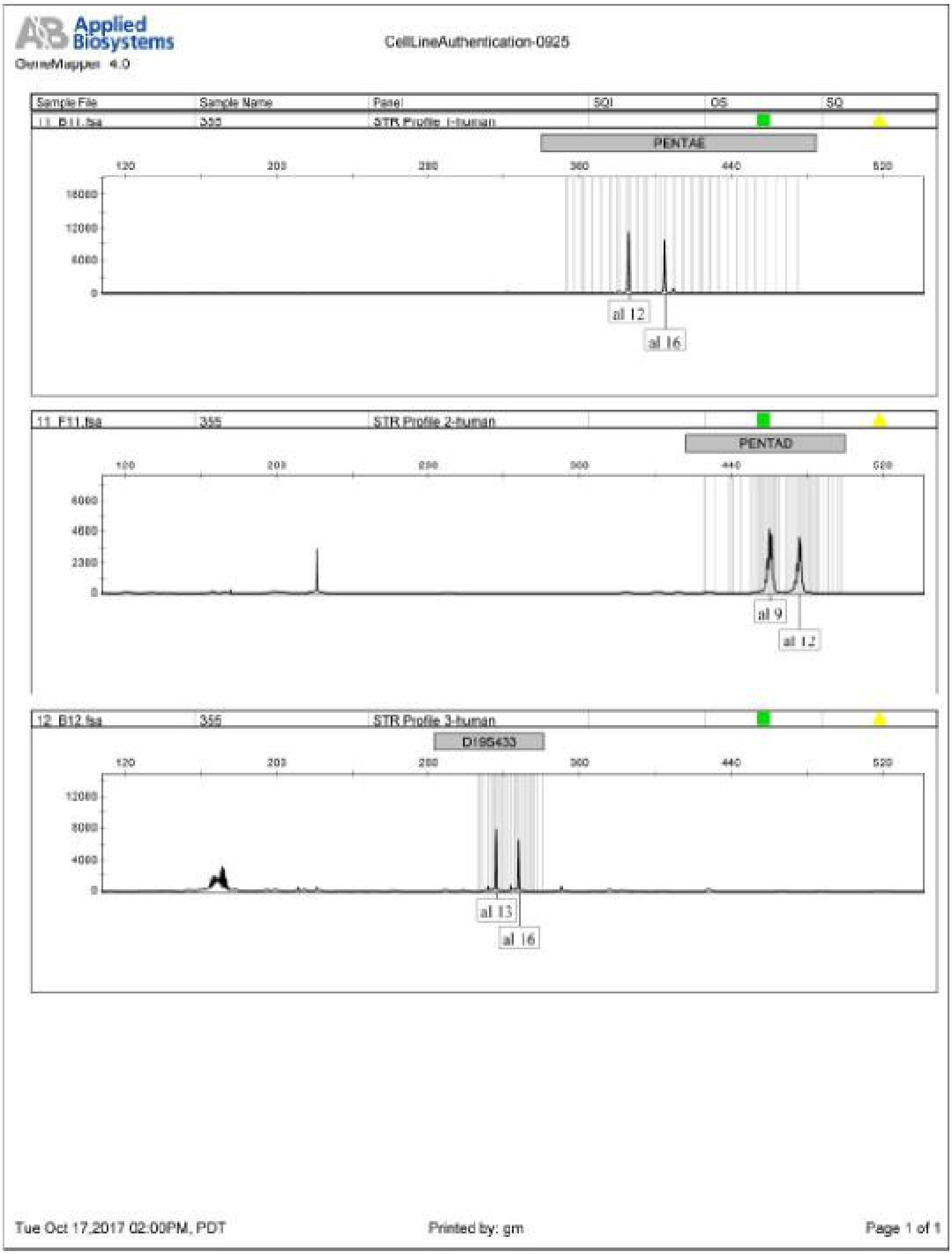

date of issue:

In September, 2017

16HBE CELL LINE TRANSFER AGREEMENT (NON-PROFIT RECIPIENT)

The 16HBE cell line (Cell Number in ATCC is CRL-2741) is being provided to you by the Provider Investigator for research purposes in your laboratory only. The material may not be distributed to any other party (for-profit or non-profit) for any reason.

Provider Investigator's Signature

[illegible] (Weidong Ji)

Recipient Investigator Signature

Yunhe

细胞遗传质量鉴定检验报告

检品名称：细胞系

检验类型：STR 基因型检验

样品编号：

表 1 样本编号

客户样本编号	公司编号
355	20170925-06

样品数量：1

样品性状：细胞系

检测项目：STR

检测方法：用 Axygen 的基因组抽提试剂盒提取 DNA，采用 20- STR 扩增方案扩增，在 ABI 3730XL 型遗传分析仪上对 STR 位点和性别基因 Amelogenin 进行检测。

检验结果：

(一) 检验基本情况

表 2：样本基因型检验结果

	多等位基因	匹配细胞系	细胞库	EV 值	匹配说明
20170925-06	无	HBE135-E6E7	ATCC	1.00	完全匹配

- 多等位基因指三等位及以上基因现象。
- 本次检测各细胞分型结果良好。

(二) 各样本描述

- 20170925-06：该株细胞 DNA 分型在细胞系检索中找到**完全匹配**的细胞系，ATCC数据库显示细胞名为 **HBE135-E6E7**，细胞号对应 **CRL-2741**。本次检测在该细胞系中**没有发现多等位基因。**

(三) 样本分型结果

细胞 20170925-06 的 STR 位点和 Amelogenin 位点的基因分型结果						
Loci	送检细胞 STR 信息			细胞库细胞 STR 信息		
	送检细胞名：355			细胞库细胞名：HBE135-E6E7		
	Allele1	Allele2	Allele3	Allele1	Allele2	Allele3
D5S818	11	12		11	12	
D13S317	8	11		8	11	
D7S820	9	10		9	10	
D16S539	9	12		9	12	
VWA	16	17		16	17	
TH01	5	6		5	6	
AMEL	X	Y		X	Y	
TPOX	8	10		8	10	
CSF1PO	9	11		9	11	

D12S391	19	24				
FGA	24	25				
D2S1338	16	17				
D21S11	28	30				
D18S51	12	12				
D8S1179	13	14				
D3S1358	15	17				
D6S1043	13	13				
PENTAE	12	16				
D19S433	13	16				
PENTAD	9	12				

其他说明：

(一) 分型方案及位点分布：

附表：实验方案及位点

	方案 1	方案 2	方案 3	方案 4
1	TH01	TPOX	D3S1358	AMEL

2	D12S391	VWA	D13S317	D5S818
3	D7S820	D8S1179	D6S1043	D2S1338
4	CSF1PO	PENTAD	D16S539	D21S11
5	FGA		D19S433	D18S51
6	PENTAE			

Applied Biosystems
GeneMapper 4.0

CellLineAuthentication-0925

Sample File	Sample Name	Panel	SQI	OS	SQ
11_B11.fsa	355	STR Profile 1-human			

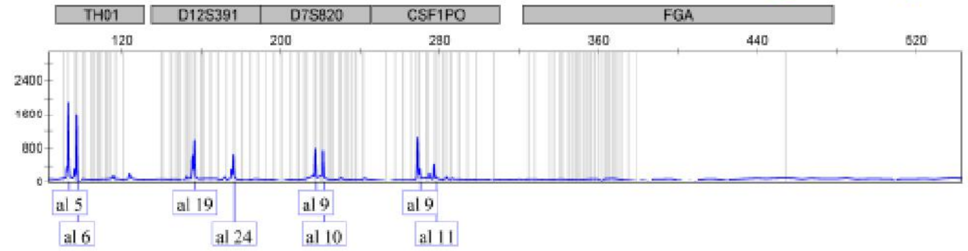

Sample File	Sample Name	Panel	SQI	OS	SQ
05_G05.fsa	355 FGA	STR Profile 1-human			

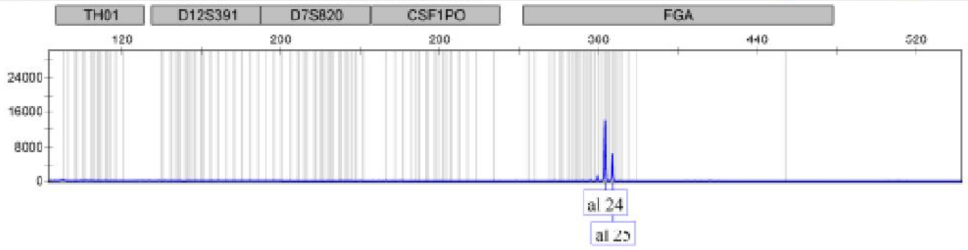

Sample File	Sample Name	Panel	SQI	OS	SQ
11_F11.fsa	355	STR Profile 2-human			

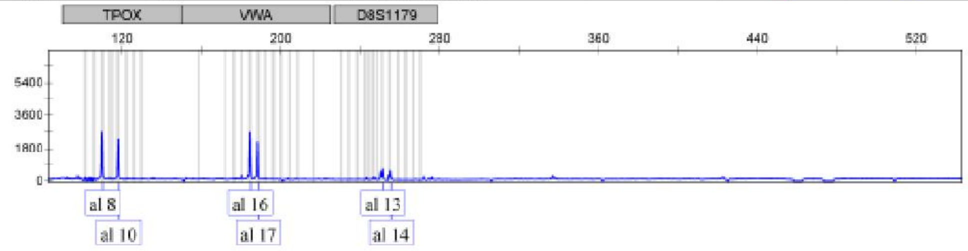

Applied Biosystems
GeneMapper 4.0

CellLineAuthentication-0925

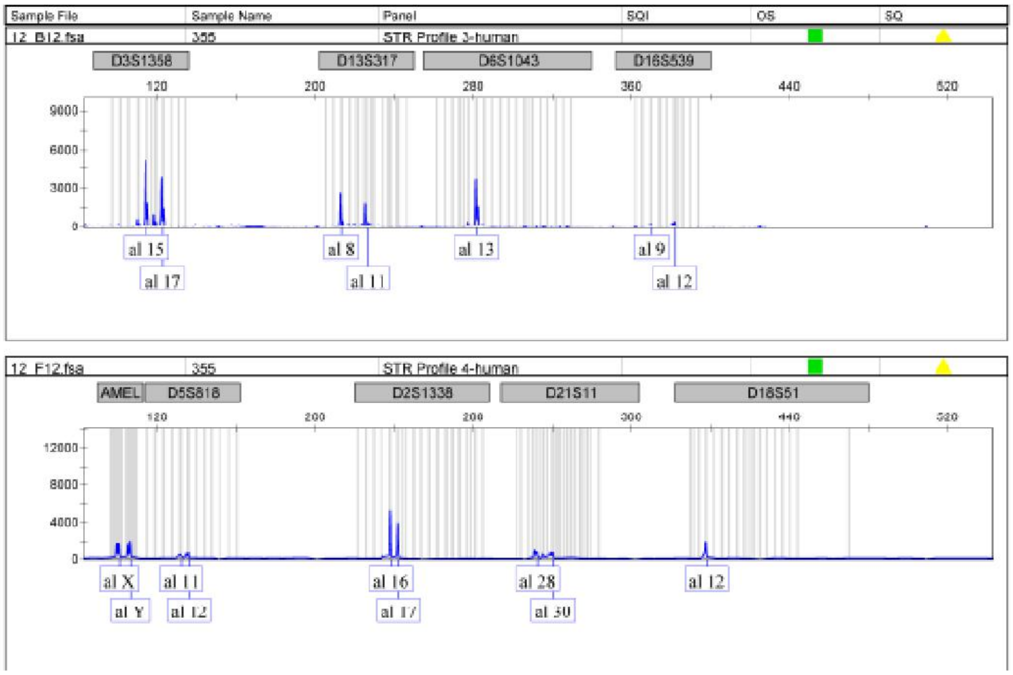

Tue Oct 17,2017 01:59PM, PDT

Printed by: gm

Applied Biosystems
GeneMapper 4.0

CellLineAuthentication-0925

Sample File	Sample Name	Panel	SQI	OS	SQ
11_B11.fsa	355	STR Profile 1-human			

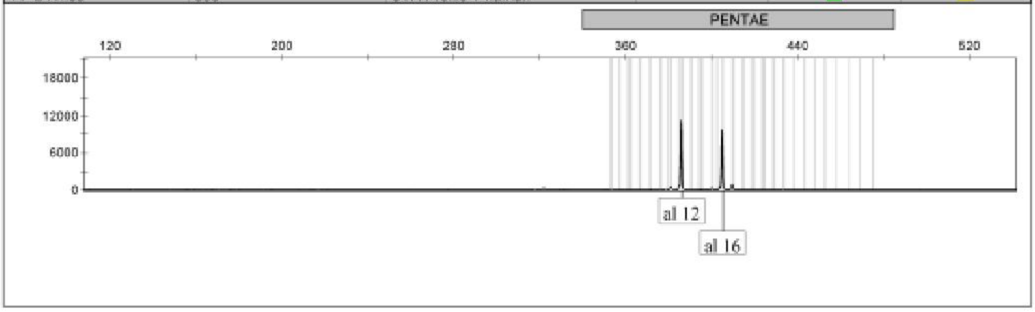

11_F11.fsa	355	STR Profile 2-human			

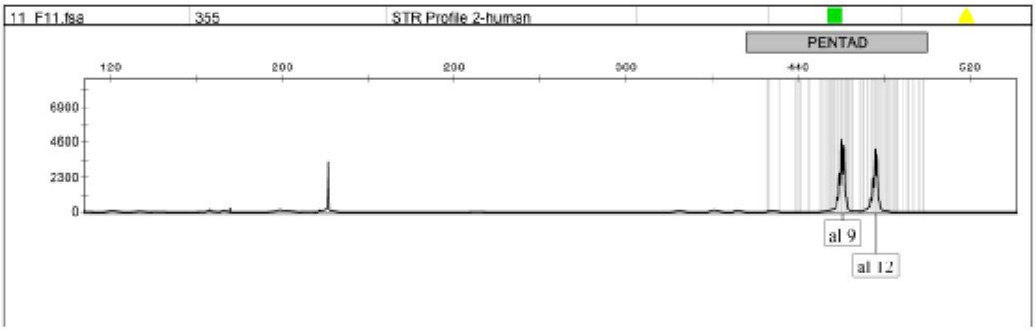

12_B12.fsa	355	STR Profile 3-human			

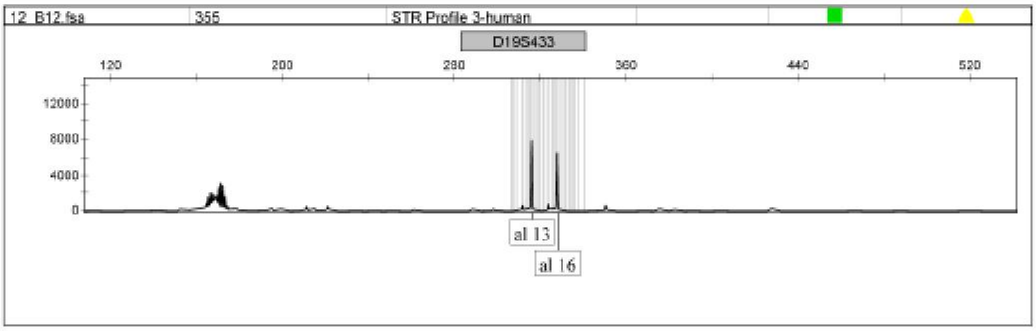

Tue Oct 17,2017 02:00PM, PDT

Printed by: gm

Page 1 of 1

签发日期：
2017 年 09 月
