# Supplementary figures and images for: Strategies for understanding the role of cellular heterogeneity in the pathogenesis of lung cancer: a cell model for chronic exposure to cigarette smoke extract
Source: BMC Pulm Med. 2022 Sep 2;22:333. doi: 10.1186/s12890-022-02116-6 (PMC9438261; doi:10.1186/s12890-022-02116-6)

支原体检测：使用依科赛生物支原体检测试剂盒with UDG PCR mix(MB00-1491S)

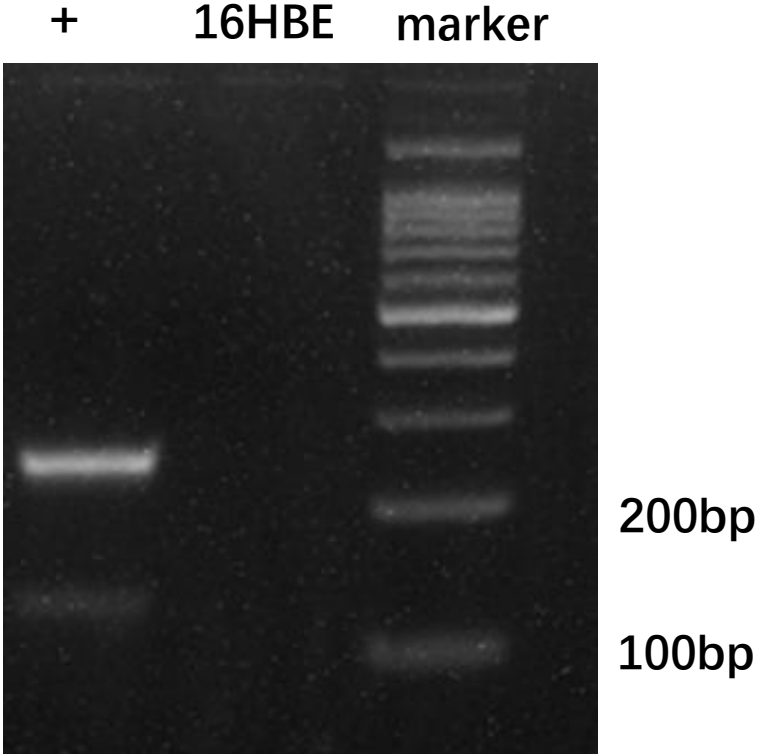

Supplement: Supplementary file 2 — Additional file 2. Growth curve date of 16HBE-B cells and 16HBE-S cells of different generations. [file 12890_2022_2116_MOESM2_ESM.pdf]
