# Supplementary material for: Strategies for understanding the role of cellular heterogeneity in the pathogenesis of lung cancer: a cell model for chronic exposure to cigarette smoke extract
Source: BMC Pulm Med. 2022 Sep 2;22:333. doi: 10.1186/s12890-022-02116-6 (PMC9438261; doi:10.1186/s12890-022-02116-6)
Supplement: Supplementary file 10 — Additional file 10. Certificate of STR Analysis of 16HBE(HBE135-E6E7)ENCN and Transfer agreement. [file 12890_2022_2116_MOESM10_ESM.pdf]

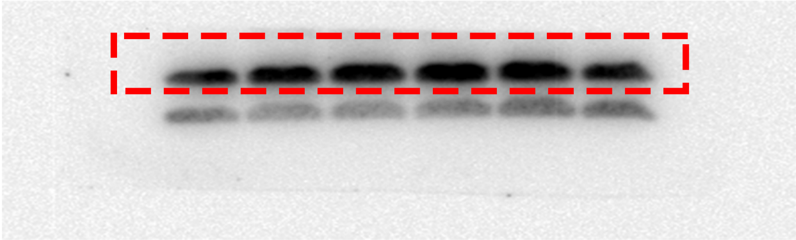

16HBE-B Control Group E-cadherin

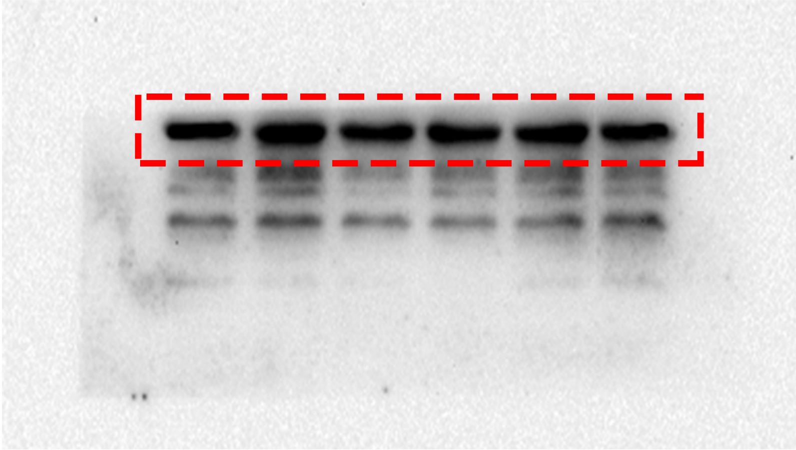

16HBE-B Control Group GAPDH

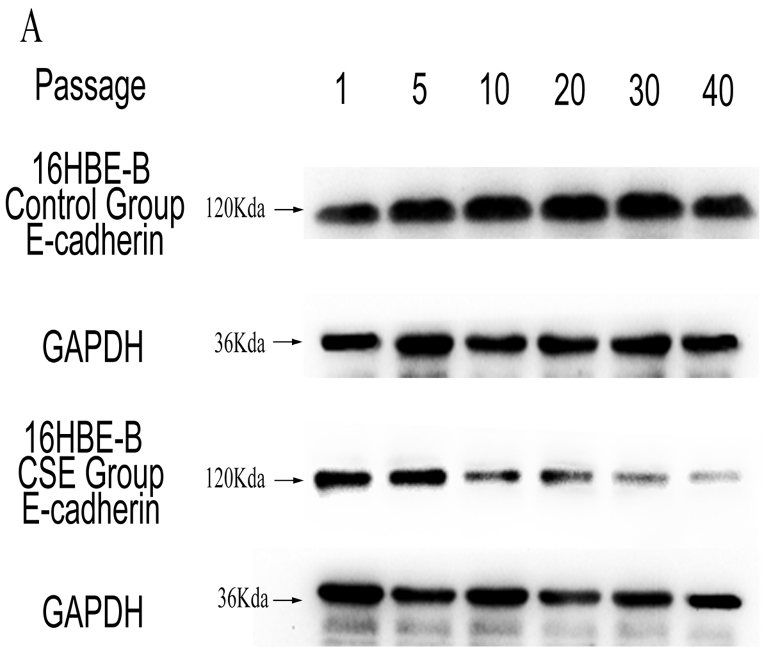

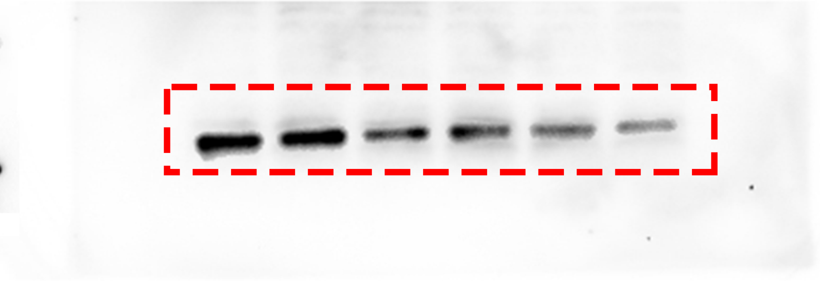

16HBE-B CSE Group E-cadherin

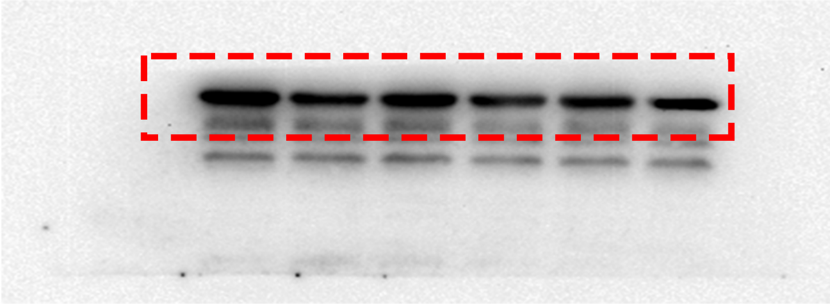

16HBE-B CSE Group GAPDH
