# Supplementary material for: Strategies for understanding the role of cellular heterogeneity in the pathogenesis of lung cancer: a cell model for chronic exposure to cigarette smoke extract
Source: BMC Pulm Med. 2022 Sep 2;22:333. doi: 10.1186/s12890-022-02116-6 (PMC9438261; doi:10.1186/s12890-022-02116-6)
Supplement: Supplementary file 11 — Additional file 11. Detection of Mycoplasmas. [file 12890_2022_2116_MOESM11_ESM.pdf]

C

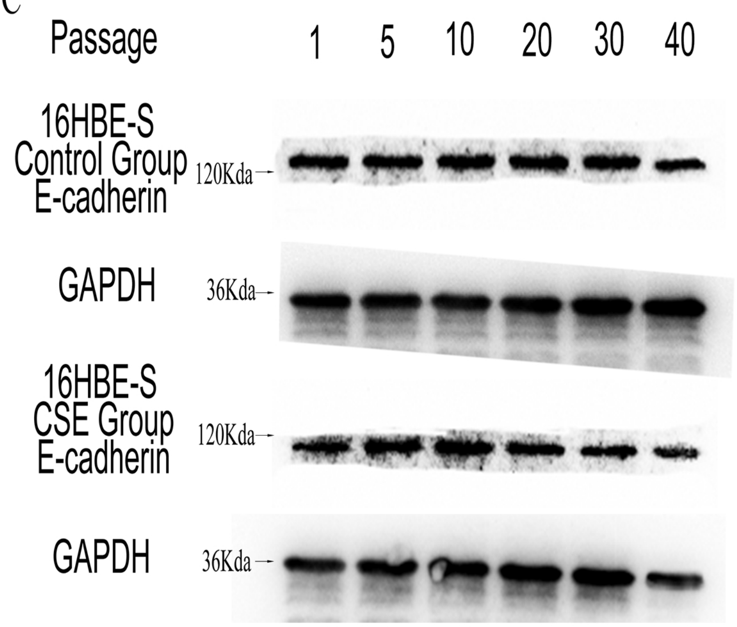

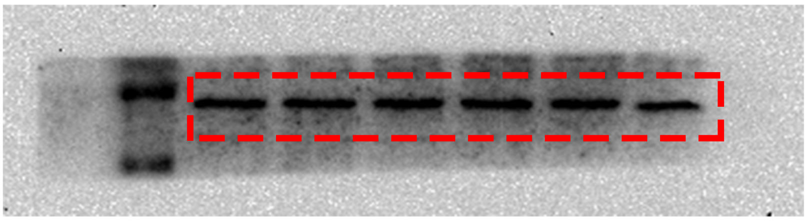

16HBE-S Control Group E-cadherin

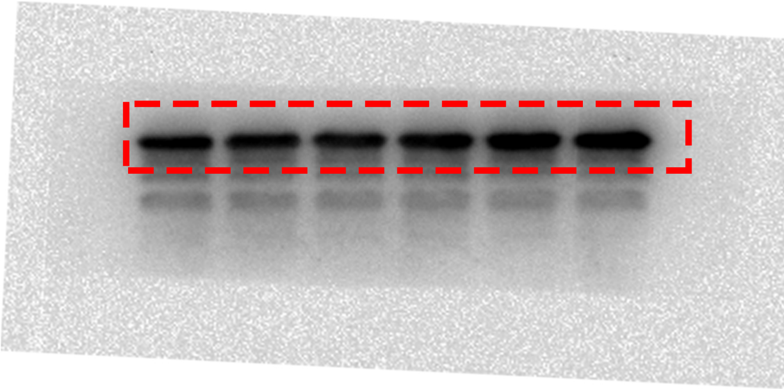

16HBE-S Control Group GAPDH

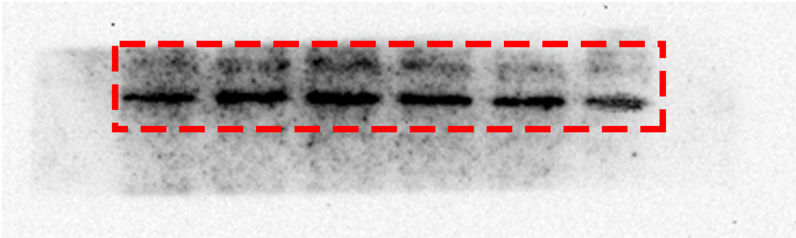

16HBE-S CSE Group E-cadherin

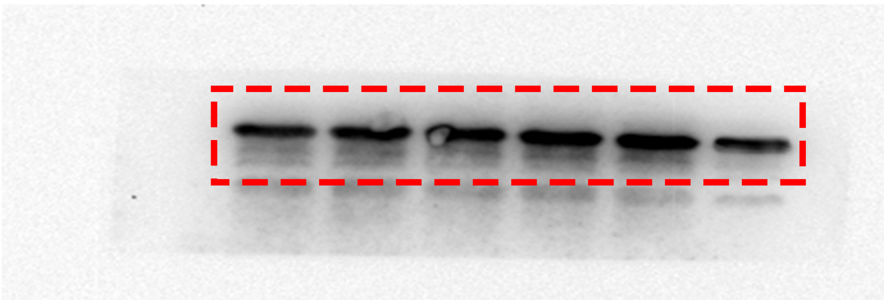

16HBE-S CSE Group GAPDH
